# Magnetic Water Cleaning of Estrogens via Tight yet Dynamic Binding to Nanoparticles

**DOI:** 10.1002/advs.77321

**Published:** 2026-09-13

**Authors:** Lukas Müller, Philip Maier, Dustin Vivod, Wenke Müller, Nikolaj Lopatik, Theresa Maria Schichtl, Julia Fröhlich, Irene Kraus, Marten Huck, Wieland Corts, Clodomiro Cafolla, Lukas Ruhm, Henrik Gaß, Johannes Voß, Jiwon Byun, Linda Rockmann, Guido Grundmeier, Kislon Voïtchovsky, Hans‐Georg Steinrück, Erdmann Spiecker, Sevim Dalabasmaz, Monika Pischetsrieder, Eike Brunner, Ralf Schweins, Dirk Zahn, Marcus Halik

**Affiliations:** ^1^ Organic Materials & Devices Institute of Polymer Materials Friedrich‐Alexander‐Universität Erlangen‐Nürnberg Interdisciplinary Center for Nanostructured Films (IZNF) Erlangen Germany; ^2^ RASEI: The CU‐Boulder/NLR Energy Institute University of Colorado Boulder Boulder Colorado USA; ^3^ Computer Chemistry Center & Chair of Theoretical Chemistry Friedrich‐Alexander‐Universität Erlangen‐Nürnberg Erlangen Germany; ^4^ Institut Laue‐Langevin DS/LSS Grenoble France; ^5^ Chair of Bioanalytical Chemistry Faculty of Chemistry and Food Chemistry Technische Universität Dresden Dresden Germany; ^6^ Food Chemistry Department of Chemistry and Pharmacy Friedrich‐Alexander‐Universität Erlangen‐Nürnberg Erlangen Germany; ^7^ Institute of Micro‐ and Nanostructure Research (IMN) & Center for Nanoanalysis and Electron Microscopy (CENEM) Friedrich‐Alexander‐Universität Erlangen‐Nürnberg Interdisciplinary Center for Nanostructured Films (IZNF) Erlangen Germany; ^8^ Institute for a Sustainable Hydrogen Economy Forschungszentrum Jülich GmbH Jülich Germany; ^9^ Institute of Physical Chemistry RWTH Aachen University Aachen Germany; ^10^ Chemistry Department Universität Paderborn Paderborn Germany; ^11^ Physics Department Durham University Durham UK; ^12^ Technical and Macromolecular Chemistry Universität Paderborn Paderborn Germany; ^13^ FAU NeW − Research Center New Bioactive Compounds Friedrich‐Alexander‐Universität Erlangen‐Nürnberg (FAU) Erlangen Germany

**Keywords:** adsorption mechanism, estrogens, micropollution, molecular interaction, self‐assembled monolayers, superparamagnetic iron oxide nanoparticles, water remediation

## Abstract

Micropollution is an ever‐increasing concern to human health and wildlife caused by population growth, industrialization, and intensive agriculture. An example of such micropollutants are natural and synthetic estrogen hormones. They are found everywhere in the aquatic environment at very low but dangerous concentrations. In this article, we demonstrate a supramolecular design principle for adsorption of such low‐concentrated estrogens onto tailored superparamagnetic iron oxide nanoparticles (SPIONs) enabling efficient magnetic water cleaning. We facilitate the adsorption by tuning the SPION surface with a binary self‐assembled monolayer (SAM) composed of two phosphonic acid derivatives: one serving as hydrophobic interaction site and the other improving water dispersibility of the system. Next to validating the concept in real river water, we conclude the demonstration of our nanomaterials by unveiling the estrogen‐SAM interaction at dilute conditions via synergistic combination of model systems, characterization techniques, and simulation. We experimentally deduce tight binding of estrogens combined with simulations that identify different, dynamic adsorption motifs at molecular space and time scale including a rare intercalation state, in which the hydrophobic pollutants minimize their contact to water. We believe that beyond our materials, these insights on the pollutant‐adsorbent interface underline the importance and potential of rational, molecular‐scale design of nano‐adsorbents.

## Graphical abstract

Micropollution is one of the big challenges humanity faces with estrogen hormones being emerging pollutants of concern. Here, a supramolecular design strategy is presented that generates a simple nano‐adsorbent material to magnetically remove low‐concentrated estrogens from water. A combination of scattering, spectroscopy, and simulation reveals the tight yet dynamic pollutant‐adsorbent binding at molecular space and time scale.

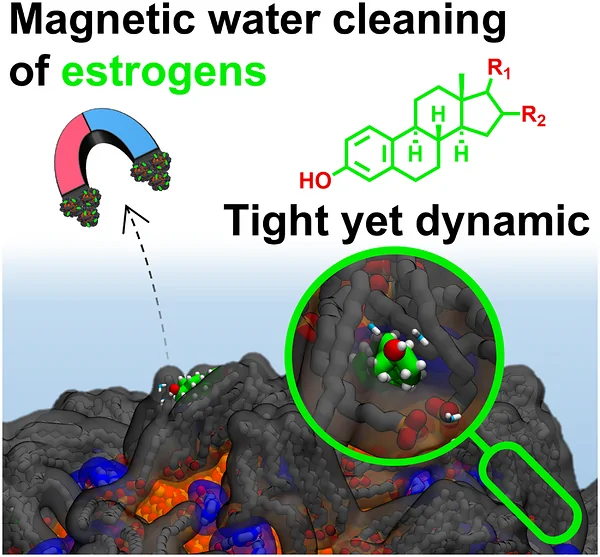

**Abbreviations**
*A, B*
: parameters
AFM: atomic force microscopy
AlO_x_
: alumina of unknown stoichiometry
ATR‐FTIR: attenuated total reflectance Fourier transform infrared
a.u.: arbitrary units
BABA: back‐to‐back
BET: Brunauer‐Emmett‐Teller
*bkg*
: incoherent background in neutron scattering data
DI: deionized
DLS: dynamic light scattering
DQ: double quantum
DTG: first derivative of TGA
*d*
: nanoparticle diameter
*d*_shell(,i)_
: thickness of the corresponding shell (SAM) part
∆*µ_water_*
: chemical potential difference between bound and unbound state in water
E_1_
: estrone
EE_2_
: 17α‐ethinylestradiol
E_2_
: 17β‐estradiol
E_3_
: estriol
ELS: electrophoretic light scattering
*GD*_PA_
: grafting density of a phosphonic acid
GIWAXS: grazing incidence wide‐angle X‐ray scattering
HR‐TEM: high‐resolution transmission electron microscopy
*I*
: scattering intensity
*k*_B_
: Boltzmann constant
MAS: magic angle spinning
MD: molecular dynamics
*MW*_PA_
: molar mass of a phosphonic acid
*n*
: number of replicates
*N*_A_
: Avogadro's constant
PAC_3_Imi+: 1‐methyl‐3‐(propylphosphonic acid) imidazolium bromide
PAC_18_
: n‐octadecylphosphonic acid
PAC_3_Imi+&PAC_18_
: binary SAM of PAC_3_Imi+ and PAC_18_ co‐self‐assembled on a surface
PAC_3_Imi+/PAC_18_
: binary SAM of PAC_3_Imi+ and PAC_18_ iteratively self‐assembled on a surface
PCB: polychlorinated biphenyl
*P*_model_
: form factor in respective model for fitting neutron scattering data
*q*
: value of the scattering vector
*ρ*
: bulk density
*r*_core_
: assumed radius of superparamagnetic iron oxide nanoparticle
SAED: selected area electron diffraction
SAM: self‐assembled monolayer
SANS: small angle neutron scattering
*SLD*_(i)_
: neutron scattering length density of the corresponding model part
sMRM: scheduled multiple reaction monitoring
SPION: superparamagnetic iron oxide nanoparticle
SQ: single quantum
*SSA*
: weight‐specific surface area
ss‐NMR: solid state nuclear magnetic resonance
TGA: thermogravimetric analysis
TiON: titania nanoparticle
*T*
: temperature
UHPLC‐DAD: ultra high performance liquid chromatography coupled to a diode array detector
UHPLC‐MS^2^
: ultra high performance liquid chromatography coupled to tandem mass spectrometry
*V_i_*
: volume accessible to estrogens in bound or unbound state i
*wl*
: relative mass loss between 150 and 700°C
WWTP: waste water treatment plant
XPS: X‐ray photoelectron spectroscopy
XRR: X‐ray reflectivity
$\chi_{\mathrm{red}}^{2}$
: reduced sum of the normalized and squared residuals

## 1 Introduction

Our water bodies and reserves are under ever‐increasing pressure due to climate change, pollution, and biodiversity loss, which are entangled dangers summarized as the “triple planetary crisis” [1, 2]. Clean water being an integral part of the 2030 agenda for sustainable development [3], the *United Nations Environment Programme* recently called for fast and focused action in regard of pollution assessment and mitigation as well as water remediation [4]. Especially anthropogenic chemical water pollution is a global concern due to micropollutants of diverse nature (heavy metal ions, micro‐ and nanoplastics, molecular pollutants etc.) having co‐dependent influence on aquatic wildlife and human health [5, 6, 7]. Such molecular pollutants are typically found at low but hazardous concentrations in the aquatic environment, although still being inadequately monitored as of 2025 [8]. The *Lancet Commission on Pollution and Health* raised serious concern on these emerging pollutants including endocrine disruptors such as hormones [9]. Representatively, this study focuses on three natural estrogens (estrone, E_1_; 17β‐estradiol, E_2_; estriol, E_3_), which play a crucial regulatory role stimulating growth, blood flow, and water retention in sexual organs [10]. Additionally, we investigate one synthetic derivative (17α‐ethinylestradiol, EE_2_) that has been employed in many oral contraceptives [11]. Excreted by humans and animals, such molecules find their way into water around the world accelerated by population growth, intensive animal agriculture and yet insufficient sewage treatment [12, 13, 14, 15, 16]. Documented estrogen concentrations in European environmental water samples typically range from 0.1 to 10 ng L^−1^ [17], while average annual quality standards in the single‐digit pg L^−1^ range have been suggested [18]. The synthetic, strongly potent EE_2_ was even found in hot spots in China at up to 17 µg L^−1^, next to being detected in remote places such as Antarctica [19]. This environmental estrogen pollution has been linked to adverse effects on aquatic life and human health including reduced fertility, feminization, as well as increased risk for developmental abnormalities and multiple cancer types, among other effects [14, 20, 21]. Since larger wastewater treatment plants (WWTPs) have to address such molecular pollutants by 2045 in the European Union [22], there is a huge demand for cost‐efficient water cleaning methods that can remove a broad range of micropollutants efficiently. Research on such methods can be categorized into biological, chemical, and physical removal approaches [23, 24]. Estrogen degradation by microorganisms, such as strains of *Sphingomonas* bacteria [25], or *ex vivo* by natural enzymes, such as laccase [26], are generally regarded as environmentally‐friendly, but relatively slow and sometimes limited for synthetic derivatives [23]. Chemical (advanced) oxidation processes, such as treatment with hydrogen peroxide or ozonation, are much faster and more efficient [27]. Therefore, such processes were already tested in German WWTPs, however, they are yet limited by severe running costs and potential toxic degradation by‐products [28, 29]. Physical removal via membrane filtration or adsorption onto carrier materials forms the third removal category. Since size exclusion filtration of estrogens is inherently limited due to its pressure requirements [23], adsorptive segregation onto large surface area providing materials, such as metal‐organic frameworks [30], molecularly imprinted polymers [31] or activated carbon [32], is more promising. The latter has also been tested in German WWTPs with still significant operating costs and limited pollutant adaptability as major drawbacks [28, 29]. One way to improve adsorptive water cleaning is simplifying the adsorbent separation after treatment, e.g., by magnetically attractable carrier materials, such as superparamagnetic iron oxide nanoparticles (SPIONs) [33, 34]. We previously introduced simple self‐assembled monolayer (SAM) coated SPIONs as sustainable, recyclable, and easily tailorable platform materials for magnetic water cleaning [35, 36, 37, 38, 39, 40]. By varying the SAM‐provided interaction functionalities, we reported removal of simple hydrocarbons including crude oil [35, 36], micro‐ and nanoplastics of various kinds and sizes [36, 37, 38], organic dyes [39], as well as persistent organic pollutants like polychlorinated biphenyls (PCBs) [40] from water. In general, the adsorption efficiency and water cleaning performance were systematically linked to the non‐covalent interplay at the pollutant‐adsorbent interface. Thus, future advancements in adsorbent design were proposed to mechanistical understanding and disentanglement of the local molecular interactions [34, 41, 42].

**FIGURE 1:**
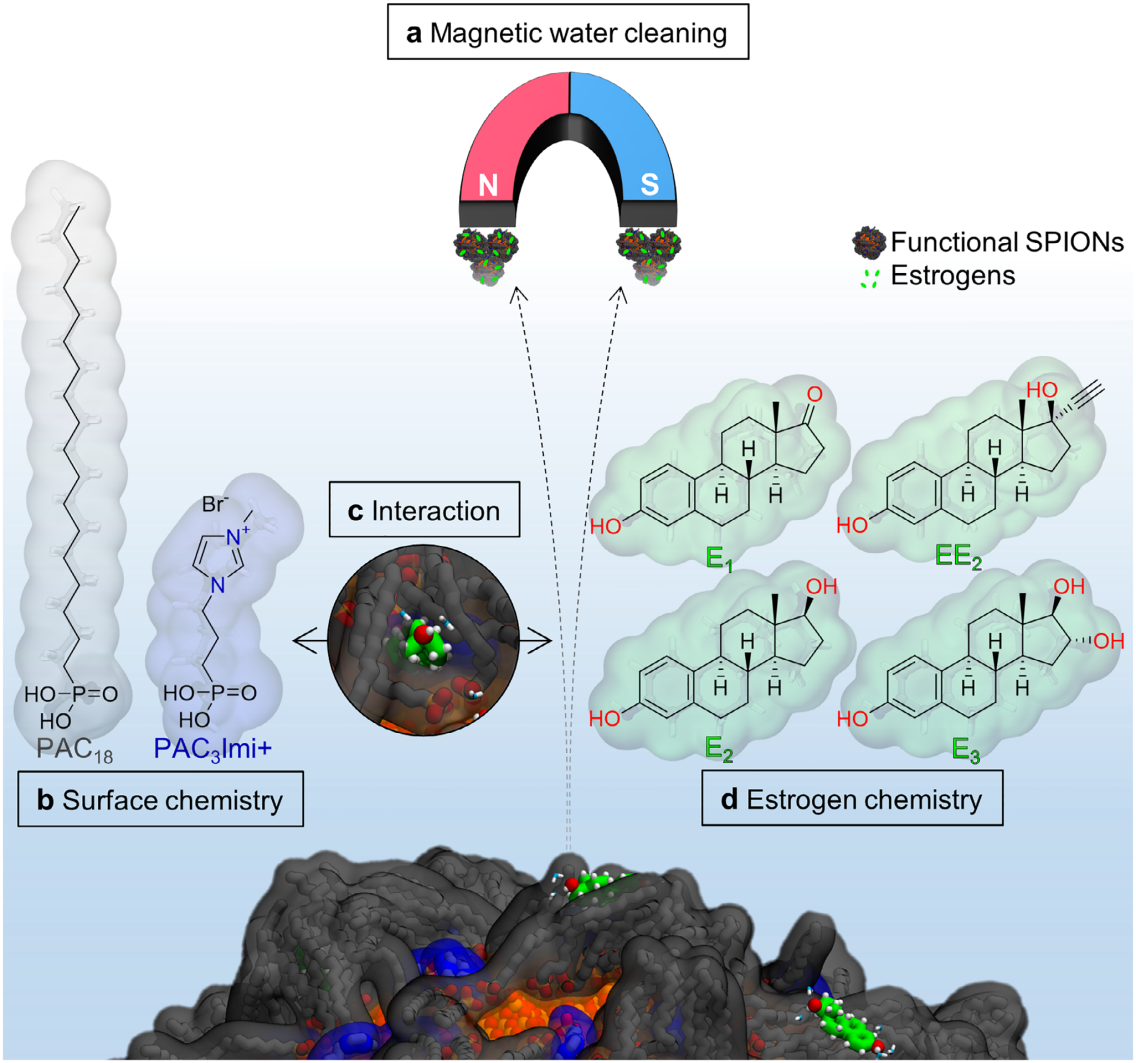
Illustration of magnetic water cleaning of estrogens via binary SAM‐coated SPIONs. a, By adsorbing the estrogens to the functional SPION surface, the water can be cleaned by simply applying a magnet so that the SPIONs are removed from the water phase carrying the pollutants along. b–d, Therefore, the surface chemistry on the SPIONs is tuned with binary SAMs composed of PAC_18_ and PAC_3_Imi+ (b) in order to provide favorable non‐covalent interaction sites (c) attracting the various estrogen derivatives E_1_, EE_2_, E_2_, and E_3_ (d) out of the water phase. The depiction of the estrogen‐SAM interface at the bottom stems from MD simulation in water and illustrates the complexity of the adsorbate‐adsorbent interaction: PAC_18_ chains are displayed in grey and PAC_3_Imi+ chains in dark blue with yellow phosphorus, red oxygen, and dark blue nitrogen atoms. The estrogens represented by E_2_ are shown with a bright green steroid body with red oxygen and white hydrogen atoms. Estrogen‐coordinating water molecules are shown with light blue oxygen and white hydrogen atoms. The iron oxide surface is shown with orange iron and red oxygen atoms.

In this article, we demonstrate a design strategy towards targeted magnetic water cleaning of low‐concentrated E_1_, EE_2_, E_2_, and E_3_ from deionized (DI) as well as sampled river water via adsorption to binary SAM‐coated SPIONs with mechanistical insights on the molecular interaction as illustrated in Figure 1. This binary SAM is composed of two strongly anchoring [43] phosphonic acid derivatives: n‐octadecylphosphonic acid (PAC_18_) that is shown to serve as hydrophobic interaction site, as well as a small fraction of 1‐methyl‐3‐(propylphosphonic acid) imidazolium bromide (PAC_3_Imi+) mainly for improved water dispersibility. The estrogen adsorption is characterized at experimentally‐challenging, low concentrations close to realistic conditions. Synergistic combination of small angle neutron scattering (SANS), solid state nuclear magnetic resonance (ss‐NMR) spectroscopy, and molecular dynamics (MD) simulations reveals a tight but diverse and dynamic non‐covalent binding of estrogens to the binary SAM. This multi‐technique approach is inspired from state‐of‐the‐art investigation of binary SAMs on (gold) nanoparticles [44] and to the best of our knowledge unprecedented in adsorbent design for water remediation. Beyond the presented functional SPIONs, we believe that these insights on pollutant‐adsorbent interaction are transferable to surface design in general and may facilitate the creation of next‐generation nano‐adsorbent materials.

## 2 Functional SPIONs Characterization

The commercial SPIONs are composed of maghemite (γ‐Fe_2_O_3_) and have an average primary diameter of about 10.7 nm calculated from their Brunauer‐Emmett‐Teller (BET) specific surface area (*SSA*) of 115.1 m^2^ g^−1^ assuming sphericity [40]. Their superparamagnetic properties were determined previously via vibrating sample magnetometer superconductive quantum interference device measurements showing a saturation magnetization of 71 emu g^−1^ [45]. They show a ζ‐potential of 1.7 ± 4.1 mV in aqueous dispersion at pH 7, at which for all presented particle systems up to µm‐scaled agglomerates are found ensuring easy magnetic collection. The full characterization of the nanoparticles comprising high‐resolution transmission electron microscopy (HR‐TEM), selected area electron diffraction (SAED), N_2_ gas adsorption, dynamic and electrophoretic light scattering (DLS/ELS) in aqueous dispersion at various pH values, as well as attenuated total reflectance Fourier transform infrared (ATR‐FTIR) spectroscopy is summarized in Figure S1. These SPIONs were covalently functionalized with PAC_3_Imi+ and PAC_18_ as well as an iteratively assembled binary SAM composed of both molecules (PAC_3_Imi+/PAC_18_). Similar SAM‐coated SPIONs behave non‐toxic in cell tests and, thus, are suitable to be used as environmental adsorbents [37, 46]. The successful SAM formation was confirmed and quantified for all systems via ATR‐FTIR spectroscopy as well as thermogravimetric analysis (TGA) combined with discussion of its implications on colloidal properties as measured by DLS/ELS given in Figures S2–S4. PAC_18_@SPIONs have a saturated phosphonic acid grafting density (*GD*_PA_) of 2.4 ± 0.2 nm^−2^ while PAC_3_Imi+@SPIONs show a lower saturated *GD*_PA_ of 1.2 ± 0.1 nm^−2^ due to repulsion of the positive charges. This translates to ζ‐potentials in aqueous dispersion at pH 7 of ‐18.7 ± 4.8 mV and 2.4 ± 1.9 mV, respectively. The composition of the binary SAM in PAC_3_Imi+/PAC_18_@SPIONs can be estimated via an ATR‐FTIR spectroscopy approach [47] that refers absorbances of CH_3_ and CH_2_ vibrations to each other resulting in about 15% of PAC_3_Imi+ and 85% of PAC_18_ (±4%) on the SPION surface. Thus, an overall *GD*_PA_ of 1.8 ± 0.1 nm^−2^ is determined, which is in between the individual saturated densities. Notably, the ζ‐potential in aqueous dispersion at pH 7 of ‐2.3 ± 2.3 mV is much closer to PAC_3_Imi+@SPIONs despite the relatively small surface share of PAC_3_Imi+. This can be systematically confirmed when (instead of self‐assembling both phosphonic acid derivatives iteratively in two steps) both derivatives are simultaneously co‐self‐assembled on the SPION surface with varying molar ratios in the assembly solution (see Figures S5–S8 and Table S1). In the resulting binary SAMs (PAC_3_Imi+&PAC_18_@SPIONs) PAC_18_ is strongly overrepresented on the surface. From an equimolar assembly solution, a surface composition of about 7% of PAC_3_Imi+ and 93% of PAC_18_ (± 7%) is generated. Nonetheless, for all surface compositions the ELS‐derived ζ‐potentials at pH 7 as well as isoelectric points are in the range of PAC_3_Imi+@SPIONs, thus confirming the dominating role of PAC_3_Imi+ on the colloidal properties.

## 3 Performance as Magnetic Estrogen Adsorbents

**FIGURE 2:**
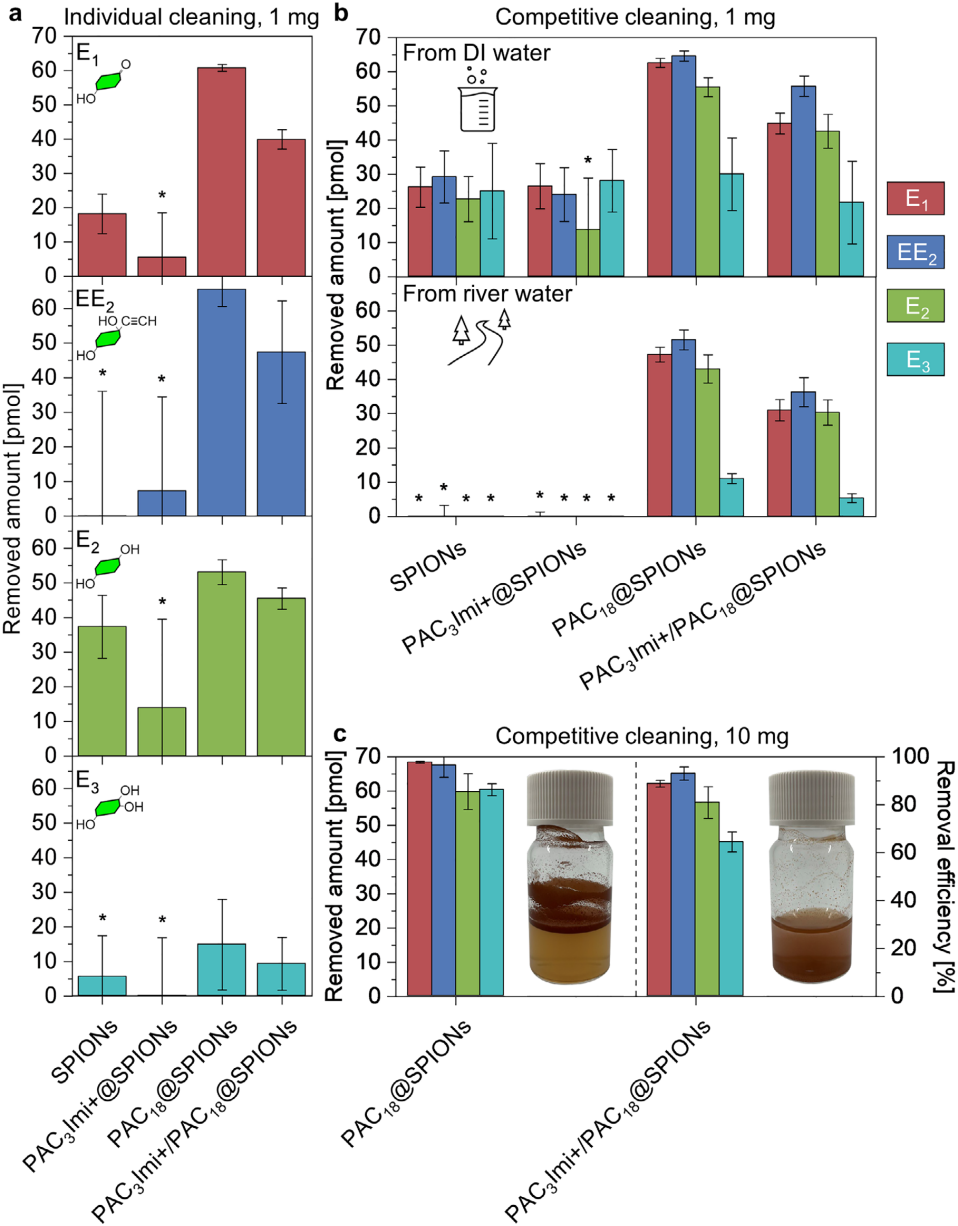
Magnetic water cleaning performances of functional SPIONs for all four estrogens. a, Removed amounts are given from treatment of aqueous solutions containing initially 70 pmol of single estrogens (7 mL à 10 nm at pH 7) with 1.00 ± 0.03 mg of functional SPIONs. b, In addition, estrogen mixtures in DI as well as river water matrices containing all four derivatives initially at 70 pmol each were treated. c, Lastly, proof‐of‐concept cleaning efficiencies were determined by treating such estrogen mixtures with 10.0 ± 0.1 mg of functional SPIONs including photographs demonstrating the improved water dispersibility of PAC_3_Imi+/PAC_18_@SPIONs. The estrogens are ordered by their hydrophobicity (E_1_>EE_2_>E_2_>>E_3_) supported by their most important structural differences displayed via pictograms. Overall, hydrophobic interactions are derived as major adsorption driver facilitated through PAC_18_ that enables removal even from river water. The most critical, synthetic EE_2_ is removed better than the hydrophobicity trend suggests. PAC_3_Imi+ does not serve as measurable interaction site, however, strongly increases water dispersibility in PAC_3_Imi+/PAC_18_@SPIONs without significantly reducing the cleaning performance. If the determined removal amounts are not at least one standard deviation apart from no removal, the data are marked with an asterisk (*) and interpreted as no significant removal. Data are given as mean ± standard deviation from *n*  =  3 (a,c) as well as *n*  =  6 (b).

To investigate the interplay of the SAM‐provided surface moieties with the four estrogen derivatives and assess the water cleaning performances, we treated estrogen‐spiked solutions (7 mL of water with 1 vol% of methanol containing 70 pmol of each estrogen at pH 7) with 1.00 ± 0.03 mg of the respective SPION system. We found this ratio well‐suited to deduce structure‐performance conclusions. The cleaning performance was evaluated by quantifying the estrogen content before and after SPION treatment via standard‐free ultra‐high performance liquid chromatography coupled to tandem mass spectrometry (UHPLC‐MS^2^) in scheduled multiple reaction monitoring (sMRM) mode. The illustrated cleaning process, sMRM parameters, photographs of all SPION dispersions before and after magnetic collection, as well as representative chromatograms and signal‐concentration correlations can be found in Figures S9–S12 and Table S2. All cleaning performances involving treatment of all four estrogens individually as well as competitively in mixtures from DI and river water (Regnitz in Erlangen, Germany) are summarized in Figure 2. Starting with the treatment of single estrogen‐spiked samples (Figure 2a), all estrogens can be collected in principle. A trend in the removed amount corresponding to the pollutant hydrophobicity (E_1_>EE_2_>E_2_>>E_3_) as ordered by their reversed‐phase chromatography retention times is obtained (see Figure S11). With 1.00 ± 0.03 mg of the hydrophobically coated PAC_18_@SPIONs 60.8 ± 1.0 pmol of E_1_, 65.5 ± 5.0 pmol of EE_2_, 53.1 ± 3.6 pmol of E_2_ and 14.8 ± 13.1 pmol of E_3_ are removed from 70 pmol containing solutions in a single treatment. On the other hand, with PAC_3_Imi+@SPIONs no significant removal is obtained and with unfunctionalized SPIONs only E_1_ and E_2_ are removed to some extent. PAC_3_Imi+/PAC_18_@SPIONs show a cleaning trend similar to PAC_18_@SPIONs, however, with reduced efficiency, especially for E_1_. When applying the functional SPIONs to aqueous mixtures of all four estrogens (70 pmol each), overall, a significant increase in cumulative removal amount is found (Figure 2b). PAC_18_@SPIONs work overall best and remove the synthetic EE_2_ with 64.6 ± 1.5 pmol slightly better than E_1_ with 62.6 ± 1.3 pmol otherwise following the above‐mentioned hydrophobicity trend. The same applies to PAC_3_Imi+/PAC_18_@SPIONs, with which the preferred removal of EE_2_ (55.7 ± 3.0 pmol) over E_1_ (44.8 ± 3.0 pmol) is even more prominent. Neither PAC_3_Imi+@SPIONs nor unfunctionalized SPIONs show a distinguishable interaction among the four estrogen derivatives with generally lower performance for all derivatives except E_3_. Interestingly, E_3_ is overall better removed in this competitive scenario than when treated individually. In challenging river water, only PAC_18_@SPIONs and PAC_3_Imi+/PAC_18_@SPIONs show adsorption and, thus, removal of the estrogens following the previously described trend with slightly reduced efficiencies compared to DI water matrices. When increasing the treatment mass of PAC_18_@SPIONs and PAC_3_Imi+/PAC_18_@SPIONs to 10.0 ± 0.1 mg, in sum about 256 and 229 pmol of in total 280 pmol of estrogens can be removed (Figure 2c). This equals overall cleaning efficiencies of 91% and 82%, respectively, including peak efficiencies of 97.8 ± 0.4% for E_1_ with PAC_18_@SPIONs and 93.0 ± 2.7% for EE_2_ with PAC_3_Imi+/PAC_18_@SPIONs in single treatments. Notably, the largest gain in efficiency is observed for E_3_, which is the least hydrophobic derivative.

The removal comparisons between the four estrogens reveal a majorly hydrophobic interaction driven adsorption on the SPIONs. The synthetic EE_2_ is removed slightly better than this hydrophobicity trend would suggest, which is beneficial due to its special environmental concern. This might be due to the additional acetylene moiety, which could facilitate hydrophobic segregation. PAC_18_ provides the hydrophobic interaction sites through long alkyl chains that enable removal even from complex river water, while no sufficiently strong interaction between the estrogens and PAC_3_Imi+ or the iron oxide surface can be concluded. However, already the small fraction of PAC_3_Imi+ in the binary SAM of PAC_3_Imi+/PAC_18_@SPIONs drastically increases their water dispersibility as shown in the photographs in Figures 2c, S10, and S13 without significantly sacrificing the overall cleaning performance. This increased dispersibility while maintaining the hydrophobic interaction sites will be necessary in large application beyond the laboratory, where homogeneous distribution in the water phase is crucial. A general increase in removal is observed from mixtures (most pronounced for E_3_), which might point to cohesion effects between derivatives underlining the importance of pollutant co‐effects and the necessity of investigating pollutant mixtures. The functional SPION surfaces are non‐saturated at the investigated pollutant concentrations and adsorbent masses (estimation between 0.001 and 0.1 estrogens per nm^2^, see Supporting Information p. 18), which is supported by successful treatment of 100x higher concentrated estrogen solutions (see Figures S14‐S16). Overall, these results clearly demonstrate the potential of the presented functional SPIONs for removal of low‐concentrated estrogens even from complex river water and suggest hydrophobic interactions as major adsorption driver.

## 4 Characterization of the Estrogen–SAM Interface

Since advancement in adsorbent design is driven by molecular scale insights on the pollutant‐adsorbent interface, we investigated the interaction of the most addressed estrogen, E_2_, with PAC_3_Imi+/PAC_18_ binary SAMs on various surfaces at relatively low estrogen concentrations. We synergistically combined non‐invasive SANS on dispersions of PAC_3_Imi+/PAC_18_@SPIONs in heavy water with ss‐NMR spectroscopy of PAC_3_Imi+/PAC_18_ on model titania nanoparticles (TiONs), both in interaction with E_2_ at 1 µm. Furthermore, we investigated PAC_3_Imi+/PAC_18_ on model flat alumina (AlO_x_) substrates via amplitude modulation atomic force microscopy (AFM), X‐ray reflectivity (XRR), and grazing incidence wide angle X‐ray scattering (GIWAXS).

**FIGURE 3:**
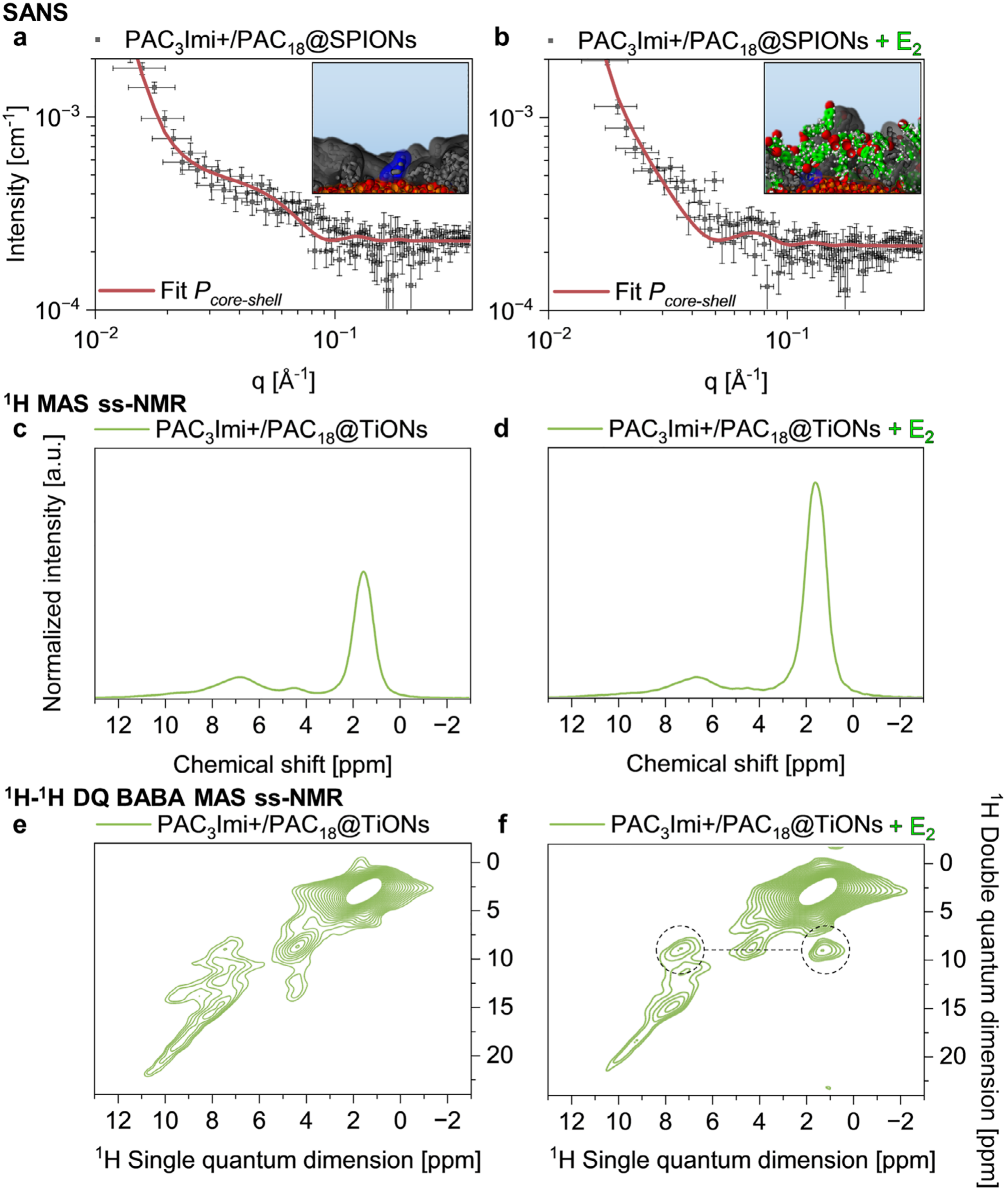
Characterization of the E_2_‐SAM interface on nanoparticle surfaces. a,b, Recorded and modeled SANS data from dispersions of 1.00 ± 0.03 mg of PAC_3_Imi+/PAC_18_@SPIONs in 7 mL of D_2_O with 1.7 vol% acetonitrile (a) and 1 µm of E_2_ additionally (b). Both scenarios are adequately fitted with a form factor model regarding spherical core‐shell SPIONs (*P*_core‐shell_). While the PAC_18_ molecules are found collapsed in a, a thickness increase of the organic shell upon adsorption of E_2_ can be deduced in b. This is illustrated by schematic inserts obtained from MD simulation. PAC_18_ chains are displayed in grey and PAC_3_Imi+ chains in dark blue with dark blue nitrogen atoms. The E_2_ molecules are shown with a bright green steroid body with red oxygen and white hydrogen atoms. The iron oxide surface is shown with orange iron and red oxygen atoms. c,d, Normalized ^1^H MAS ss‐NMR spectra of dried model PAC_3_Imi+/PAC_18_@TiONs without (c) and with adsorbed E_2_ (d). e,f, ^1^H‐^1^H DQ BABA MAS ss‐NMR spectra of the same nanoparticles without (e) and with adsorbed E_2_ (f). On the dried model TiONs, E_2_ can be identified via the increased signal intensity in d and the indicated cross‐peaks in f. The given error bars account for *q* smearing and intensity measurement uncertainty, which both were accounted for in data modeling (a,b).

The SANS patterns recorded from dispersions of 1.00 ± 0.03 mg of PAC_3_Imi+/PAC_18_@SPIONs in 7 mL of D_2_O with 1.7 vol% acetonitrile (a) and 1 µm of E_2_ additionally (b) are given in Figure 3. Despite the low nanoparticle and estrogen concentrations a significant difference in the scattering patterns can be observed. The recorded scattering intensity *I(q)* is modeled with a sum of an inverse power term of the value of the scattering vector *q* scaled with parameter *A* (Porod's law [48] accounting for SPION agglomerates), a form factor model *P*_model_ *(q)* scaled with parameter *B* (accounting for individual SPIONs) and the contribution of the incoherent background *bkg* after subtraction of solvent and empty cell scattering as given in Equation (1).

(1) $$I\;\left(q\right)=A\cdot q^{-4}+B\cdot P_{\mathrm{model}}\left(q\right)+bkg$$

We applied three form factor models: A spherical nanoparticle (*P*_core_), a spherical core‐shell nanoparticle (*P*_core–shell_) and a spherical core–shell–shell nanoparticle structure (*P*_core–shell–shell_). While the first model was employed to rationalize the core‐shell models, the latter was chosen to assess if it provides a more accurate description of the actual scattering length density (SLD) profile of the particles. An illustration of the SLD profiles, all modeling parameters including derivations as well as all model fit curves are given in Figures S17–S18 and Table S3. For all fits, *r*_core_ was fixed to 53.65 Å, which corresponds to the BET‐determined SPION radius. Overall, we found that inclusion of a shell in the form factor enables a more adequate modeling of the SANS between 0.02 and 0.2 Å^−1^. *P*_core‐shell‐shell_ yields a reasonable fit only with adsorbed E_2_ underlining its double shell‐like morphology, while the simpler *P*_core–shell_ model fits both SANS patterns sufficiently well. This is why only *P*_core‐shell_ is discussed in the following and depicted in Figure 3. The reduced sum of the normalized and squared residuals ($\chi_{\mathrm{red}}^{2}$) as well as the fitted shell thicknesses *d*_shell_ from modeling with *P*_core‐shell_ are given in Table 1. Without any E_2_ present, *d*_shell_ of PAC_3_Imi+/PAC_18_@SPIONs is estimated at 6.9 ± 2.4 Å, which is much smaller than the extended chain length of PAC_18_ molecules of about 21.7 Å. This observation suggests a collapse of these hydrophobic molecules in aqueous environment [49]. However, upon adsorption of E_2_, *d*_shell_ increases to 17.7 ± 6.3 Å due to the adsorbed E_2_ in and on the shell (estimation between 0.02 and 1.91 E_2_ molecules per nm^2^, see Supporting Information, p. 18). Simultaneously, D_2_O is pushed away from the SAM as the differences in *SLD*_in_ (see Table S3) and supporting MD simulations in Figure S19 show. Thus, the adsorption of E_2_ has a measurable influence on the organic nanoparticle shell morphology and on the interaction of SAM and water.

**TABLE 1:** Comparison of fit quality and shell thicknesses from modeling with *P*_core–shell_. Both SANS patterns, from PAC_3_Imi+/PAC_18_@SPIONs without and with adsorbed E_2_, are adequately modeled with *P*_core‐shell_ as judged by $\chi_{\mathrm{red}}^{2}$ . The adsorption of E_2_ leads to a significant shell thickness increase in the aqueous environment. All further parameters are given in Table S3. The fitted values are given with 95% confidence intervals.

Form factor model & spatial dimensions	PAC_3_Imi+/PAC_18_@SPIONs	PAC_3_Imi+/PAC_18_@SPIONs + E_2_
*P*_core‐shell_	$\chi_{\mathrm{red}}^{2}$ = 1.31	$\chi_{\mathrm{red}}^{2}$ = 1.30
*r*_core_ (fixed) [Å]	53.65	53.65
*d*_shell_ ∈ [3.0;32.6] [Å]	6.9 ± 2.4	17.7 ± 6.3

Since the functional SPIONs are not suitable for ss‐NMR spectroscopy, we representatively investigated PAC_3_Imi+/PAC_18_@TiONs. The nanoparticle cores were characterized via HR‐TEM, SAED, and N_2_ gas adsorption, while the functionalization was confirmed via ATR‐FTIR spectroscopy and TGA (see Figures S20–S22). In short, these models represent the SPIONs reasonably well with an average diameter of 17.6 nm covered with a binary SAM consisting of about 17% of PAC_3_Imi+ and 83% of PAC_18_ with systematically lower *GD*_PA_. The ^1^H magic angle spinning (MAS) ss‐NMR spectra of dried PAC_3_Imi+/PAC_18_@TiONs before and after E_2_ adsorption from 1 µm pollutant solution are given in Figure 3c,d. Note that the spectra were normalized to the sample mass to allow for the direct comparison of signal intensities. The resolution of the ^1^H spectra is not sufficient to distinguish the various ^1^H species present in the samples. Therefore, they were deconvoluted and reference spectra recorded (see Figures S23–S29). Using these reference spectra, proton signals on the binary SAM coated nanoparticles between 0 and 3 ppm can be assigned to aliphatic protons mainly originating from PAC_18_. Signals between ca. 3 and 5 ppm are primarily attributed to CH_2_ and CH_3_ groups on the imidazolium ring of PAC_3_Imi+ with some potential contribution from methanol remaining from the functionalization process and atmospheric water. Lastly, signals between ca. 6 and 10 ppm are assigned to the superimposing contributions of aromatic CH protons of the imidazolium ring, hydroxyl protons of phosphonic acid groups, and unbound hydroxyl groups on the TiONs. The most striking change caused by E_2_ adsorption is a pronounced increase of the signal intensity of aliphatic protons at ca. 1.6 ppm. This increase points towards the presence of E_2_ with its main signal around the same shift (see Figure S24). Adsorption of methanol as a reason for this effect is highly unlikely because the chemical shift of its CH_3_ amounts to ca. 3.4 ppm. To further investigate the interaction between E_2_ and the SAM, we recorded ^1^H‐^1^H double quantum (DQ) back‐to‐back (BABA) MAS ss‐NMR spectra as shown in Figure 3e,f, as well as in Figure S30. With this technique recoupling of protons in close spatial proximity (<0.35 nm) can be generated [50].

The ^1^H‐^1^H DQ BABA MAS ss‐NMR spectra before and after E_2_ loading mainly exhibit diagonal signals: An intense signal due to aliphatic ^1^H between 0 and 3 ppm, a signal at ca. 4.4 ppm due to CH_2_ and CH_3_ groups attached to the imidazolium ring, as well as weaker signals in the region between 6 and 10 ppm due to aromatic ^1^H and OH groups. Dipolar coupling between different ^1^H species would result in correlations at different shifts in single quantum (SQ) dimension and at the sum of the two shifts in DQ dimension. In Figure 3e no such intense correlations could be unequivocally detected and assigned at the given signal‐to‐noise ratio. However, a pronounced correlation between aliphatic ^1^H at 1.4 ± 0.2 ppm and aromatic ^1^H at 7.5 ± 0.2 ppm in SQ dimension (ca. 8.9 ppm in DQ dimension) is clearly visible after E_2_ loading as indicated in Figure 3f. This correlation is characteristic for the contact between aliphatic and aromatic protons within E_2_ as can be seen from the spectrum of pure E_2_ (see Figure S30a). Note that the chemical shifts of pure E_2_ are about 0.6 ppm smaller than in the adsorbed state (pure E_2_: 0.8 ± 0.2 ppm and 7.0 ± 0.2 ppm in SQ dimension; ca. 7.8 ppm in DQ dimension), which can be explained by the adsorption of E_2_ in the binary SAM. This means the ^1^H‐^1^H DQ BABA MAS ss‐NMR spectra clearly confirm the presence of significant amounts of E_2_ after loading. Other dipolar contacts than the discussed could not be detected at the given signal‐to‐noise ratio. This may be explained by preferential localization of E_2_ on the SAM surface and/or contact with the aliphatic chains of PAC_18_. The latter signal appears at a similar chemical shift as the main E_2_ signal, i.e., the signals are superimposed. This would prevent the resolution of possible correlations between E_2_ and PAC_18_. Moreover, adsorbed E_2_ may be relatively mobile, which would weaken or even suppress such intermolecular correlations (see MD simulations).

Lastly, we investigated flat PAC_3_Imi+/PAC_18_@AlO_x_ since this approximation facilitates access to properties such as SAM order and crystallinity [51, 52, 53]. However, we found that these binary SAMs on flat substrates did not offer a suitable model for the SPIONs due to their deviating composition of about 86% PAC_3_Imi+ and 14% PAC_18_ as estimated by X‐ray photoelectron spectroscopy (XPS). Nonetheless, we did find differences in SAM crystallinity between this iterative self‐assembly and the simultaneous co‐self‐assembly without being able to conclude any further influence of E_2_ on the SAM morphology. The full study in terms of water contact angles, surface energies, XPS, AFM, XRR, and GIWAXS is summarized in Figures S31–S35 and Tables S4–S6.

## 5 MD simulations Unveil Dynamic Interaction with Rare Intercalation States

**FIGURE 4:**
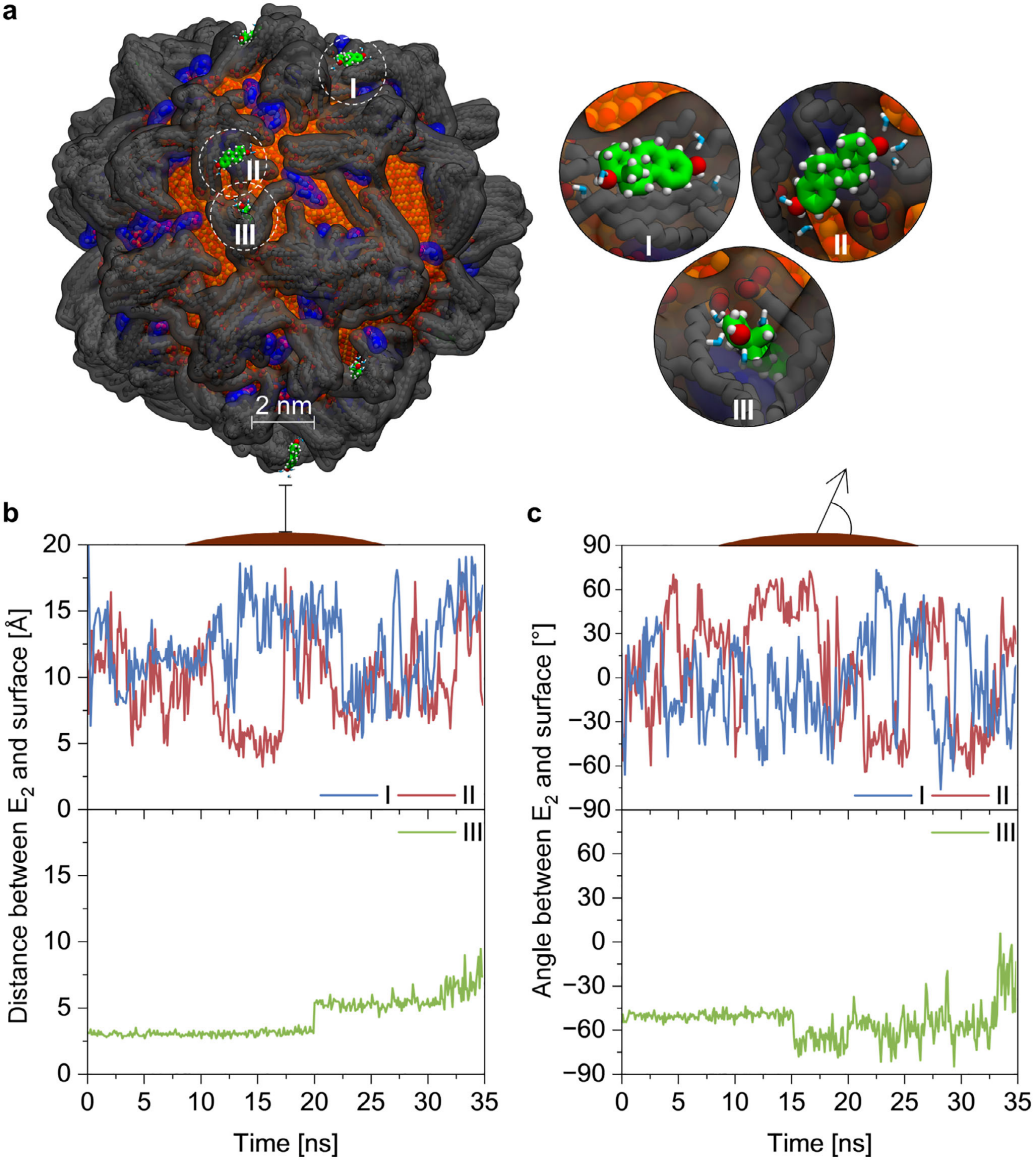
MD simulation of E_2_ in interaction with a binary SAM‐coated SPION in water. a, 10 E_2_ molecules were simulated on a PAC_3_Imi+ and PAC_18_ binary SAM‐coated SPION in water over 35 ns at 300 K and 1 atm. PAC_18_ chains are displayed in grey and PAC_3_Imi+ chains in dark blue with yellow phosphorus, red oxygen, and dark blue nitrogen atoms. The E_2_ molecules are shown with a bright green steroid body with red oxygen and white hydrogen atoms. Estrogen‐coordinating water molecules are shown with light blue oxygen and white hydrogen atoms. The iron oxide surface is shown with orange iron and red oxygen atoms. b,c, Representing different initiation scenarios (type I: top of PAC_18_ bundle; type II: side of PAC_18_ bundle; type III: intercalated in PAC_18_ bundle), three E_2_ molecules are analyzed in terms of their distance (b) and angle to the SPION surface (c). In (c) 0° means being parallel and ±90° orthogonal to the surface. Overall, all E_2_ molecules are tightly bound to PAC_18_. Types I and II are interchangeable both showing free surface diffusion along the respective PAC_18_ bundle, while for rare type III (occurrence of 9%) the E_2_ molecule stays intercalated over the whole simulation duration.

Complementary to the SANS and ss‐NMR spectroscopy experiments, we set up MD simulations of a PAC_3_Imi+ and PAC_18_ binary SAM‐coated SPION based on the respective particle characterization discussed before (see Figure S36). Pre‐screening of estrogen‐SPION association sites was performed by nanoparticle immersion into pure estrogen, followed by random selection of 3 × 10 adsorption poses. Using 3 × 4 independent runs each featuring 10 adsorbed pollutants of E_1_, EE_2_, E_2_, and E_3_, respectively, relaxation in water was investigated from 35 ns scale runs. Allowing the first 5 ns for system equilibration, we then investigated the dynamics of pollutant‐SPION binding motifs from 30 ns scale simulation runs. At 300 K all estrogen molecules remained adsorbed on the SAM over the whole simulation with most being tightly bound to PAC_18_ bundles. While no direct estrogen‐PAC_3_Imi+ contacts were observed, PAC_3_Imi+ attracts water molecules, which supports the formation of these nm‐scaled PAC_18_ bundles. The estrogen‐surface interaction in water appeared mostly dynamic rather than static. Representing different association motifs, the dynamics of three E_2_ molecules (type I: initiated on top of a PAC_18_ bundle, type II: initiated at the side of a PAC_18_ bundle, and type III: initiated intercalated in a PAC_18_ bundle) are analyzed in Figure 4 in terms of their distance and angle to the SPION surface (see Videos S1–S5). Video S1 shows the dynamics of the whole SPION, while Videos S2‐S4 show type I, II, and III motifs, respectively. Video S5 shows type III binding from another perspective. Water that does not coordinate with estrogens is not shown. In type I and II, the E_2_ molecules freely rotate and diffuse along the hydrophobic surface while retaining the water‐coordination of hydroxyl (or keto for E_1_) groups. Both deliver a similar number of hydrophobic interaction sites, which renders them interchangeable motifs. A very different behavior is observed for type III, where the E_2_ molecule stays immobilized in a PAC_18_ bundle until the end of the investigated time frame. This full submersion in hydrophobic PAC_18_ allows only one hydroxyl group of each E_2_ to interact with water molecules, while the water‐coordination of the other is replaced with contacts either with the phosphonic acid anchor groups of the SAM or the iron oxide surface. Overall, the hydrophobic contact area provided by PAC_18_ is maximized and the contact to water minimized in type III binding (see coordination analyses in Figures S37–S44 and Table S8). However, this motif is rarely observed: About 3% for E_1_, 3% for EE_2_, 9% for E_2_, and 6% for E_3_. Similar surface diffusion of amphiphilic adsorbates on SAMs with distinguishable binding motifs including rare populations was experimentally found and described before [54].

While all of the estrogen derivatives remained fully bound to the SPION in the MD runs performed at 300 K, we also subjected our model systems to high‐temperature in order to explore the free energy of pollutant binding from Boltzmann statistics. At 750 K (imposing constant volume of the simulation cells) sufficient statistics of association/dissociation events was assessed from additional 10 ns runs (sampled after 5 ns equilibration). To provide temperature‐independent differences in chemical potential ∆*µ* between bound and unbound state, we compensated the binding free energies by an −*k*_B_ *T* ln(*V*_bound_/*V*_free_) entropy term stemming from the different volumes available to estrogen molecules bound to the SPION (*V*_bound_) and unbound in bulk solution (*V*_free_), respectively (see Equation S5). *k*_B_ is the Boltzmann constant and *T* is temperature. On this basis, we find that the combination of hydrophobic segregation and suitable hydrogen bonding of the amphiphilic estrogen molecules promotes pollutant adsorption from aqueous solution by 4–4.5 kcal mol^−1^ (see ∆*µ*_water_ in Table 2). E_3_ shows the weakest adsorption trend reflecting the cleaning performances discussed before. To assess pollutant affinity upon magnetic removal out of the water phase, we also explored the association/dissociation statistics from analogous runs, but in absence of water. While hydrophobic interactions are obviously dismissed, we find reorganization in favor of estrogen‐phosphonic acid and estrogen‐iron oxide interactions. We suggest this as the mechanism how estrogens remain on the nanoparticle surface upon removal from water.

**TABLE 2:** Chemical potential differences between bound and unbound states of estrogen derivatives on a PAC_3_Imi+ and PAC_18_ binary SAM. The chemical potential differences were calculated in water from equilibria of adsorbed and desorbed estrogens. Data are given as mean ± standard deviation from three independent simulations each. Based on Boltzmann statistics, the adsorption preferences of E_1_, EE_2_, and E_2_ relative to E_3_ were derived, respectively.

Estrogen derivative	E_1_	EE_2_	E_2_	E_3_
∆*µ*_water_ [kcal mol^−1^]	−4.3 ± 0.6	−4.5 ± 0.5	−4.5 ± 0.4	−4.0 ± 0.2
Adsorption preference rel. to E_3_[‐]	3.4 ± 2.8	3.6 ± 2.8	3.2 ± 2.2	1

## 6 Conclusion

In summary, we demonstrated a supramolecular design strategy relying on simple phosphonic acid derivatives and ton‐scale produced SPIONs for magnetic water cleaning of low‐concentrated (10 nm) estrogens represented by the natural derivatives E_1_, E_2_, and E_3_ as well as by the synthetic EE_2_. We showed excellent cleaning efficiencies of up to 97.8 ± 0.4% in a single treatment from estrogen mixtures for the most hydrophobic derivative‐SAM pairing and validated the adsorption‐based approach in real river water at dilute conditions. Fair efficiency comparison to other studies is hardly possible in such low‐concentration and unsaturated‐adsorbent scenarios, which is why we call for establishing standard procedures regarding pollutant concentrations, experimental setups, and quantification approaches. We focused on elucidating the complex pollutant‐adsorbent interaction. Therefore, we combined multiple experimental techniques and model systems with simulation, since we did not find any single technique being able to unveil the diverse and dynamic interplay at molecular space and time scale under dilute conditions. We ruled out flat substrates as a suitable model for this investigation due to deviating binary SAM compositions. For SPIONs with binary SAMs of some PAC_3_Imi+ and mostly PAC_18_, we disentangled the local molecular interactions: The adsorption was found to be mainly driven by hydrophobic segregation onto PAC_18_, while PAC_3_Imi+ strongly improved water dispersibility of the magnetic nano‐adsorbents and supported the formation of nm‐scaled PAC_18_ bundles. This counterintuitive combination of hydrophobicity with water dispersibility will be important when scaling up water treatment volumes and is generally transferable to any SAM‐tunable adsorbent. Furthermore, we showed that the PAC_18_ chains collapse in aqueous environment with the subsequent adsorption of E_2_ pushing water molecules away from the surface. While this adsorption to PAC_18_ is tight, we demonstrated its diverse and dynamic nature on the particle surface. We identified a rare adsorption motif in the simulations, in which the estrogens are trapped in a hydrophobic PAC_18_ bundle minimizing their water contact. Such diversity in surface dynamics is typically disregarded in adsorbent design by only investigating macroscopic ensemble properties. We believe that overcoming such dynamics by promoted intercalation, e.g., by rational design of mixed SAMs via directional interactions with suitable sterics provided by combination of different chain lengths, could serve as a blueprint for selective and high‐performant nano‐adsorbents. Given the modular nature of SAMs, this may enable targeted removal of very low‐concentrated micropollutants from complex water matrices beyond the demonstrated estrogens.

## 7 Methods

### 7.1 Materials

The SPIONs were kindly provided by Comar Chemicals (Pty). The phosphonic acid derivatives PAC_3_Imi+ (≥95%) and PAC_18_ (≥97%) were obtained from SiKÉMIA. The estrogens E_1_ (≥99%), EE_2_ (≥98%), E_2_ (≥98%) and E_3_ (≥97%) were acquired from Sigma Aldrich. As solvents for functionalization and the estrogen removal experiments methanol (≥99%) and acetonitrile (HPLC grade) were purchased from Carl Roth. The DI water was taken from the central purification supply at the Interdisciplinary Center for Nanostructured Films, while the river water was sampled from the Regnitz in Erlangen (49°35′14.2″ N 10°58′51.9″ E) on August 12, 2024. It was allowed to settle for 24 h, decanted, filtered twice through a nylon stocking (30 DEN ‐ 33 dtex) and stored protected from light until usage. Acid and base solutions for titration were prepared from hydrochloric acid (37%) and solid sodium hydroxide (≥99%) acquired from Carl Roth. For UHPLC‐MS^2^/‐DAD quantification ultrapure water delivered by a Reference A+ Milli‐Q device from Merck Millipore, methanol in Ultra LC‐MS grade from Carl Roth, and acetonitrile in LC‐MS grade from Fisher Scientific were used. D_2_O from Innovachem (≥99.8%) for SANS experiments was provided by the Institut Laue‐Langevin. TiONs for ss‐NMR spectroscopy were in stock of the group of M. Halik but the exact supplier is unknown to the authors.

### 7.2 Nanoparticle Functionalization

For unitary SAMs, 5 mm solutions of PAC_3_Imi+ or 10 mm solutions of PAC_18_ were prepared in methanol and mixed with methanol‐based dispersions of SPIONs (3 mg mL^−1^) at a volumetric ratio of 3:10, respectively. These concentrations were previously determined as minimal to yield saturated SAMs via concentration series. The mixtures were sonicated for 30 min at 25°C in a Sonocool 255 from Bandelin followed by sedimentation via centrifugation with a Multifuge X1R from Heraeus at 13,000 rpm and 5°C (30 min for PAC_3_Imi+ and 15 min for PAC_18_). Subsequently, the particles were washed twice to remove unbound phosphonic acids, where each iteration consisted of redispersing them in the initial volume of fresh methanol, 10 min of sonication and sedimentation via centrifugation at 13,000 rpm and 5°C (same duration as before). For assembly of iterative binary PAC_3_Imi+/PAC_18_ SAMs, the whole process was first conducted with PAC_3_Imi+ followed by a repetition with PAC_18_. To obtain co‐assembled binary PAC_3_Imi+&PAC_18_ SAMs, 10 mm PAC_3_Imi+ and PAC_18_ solutions were mixed in variable ratios prior to giving them to the SPION dispersions. For TiONs the processes were carried out identically. Finally, the functionalized particles were dried at 60°C over‐night.

### 7.3 Nanoparticle Characterization

For HR‐TEM and SAED, methanol‐based nanoparticle dispersions (0.5 mg mL^−1^) were drop‐cast onto 400 mesh ultrathin lacey carbon Cu grids. Measurements were performed at 300 kV using a Thermo Fisher Scientific double‐corrected Titan Themis^3^ 300 microscope equipped with a double‐tilt holder. Data acquisition and processing were carried out using Velox software also from Thermo Fisher Scientific. Background subtraction and peak fitting of the rotationally averaged SAED patterns were performed using LIPRAS [55]. For identification of the respective crystal phase, diffraction patterns were compared with entries in the Crystallography Open Database using JEMS software [56].

N_2_ gas adsorption measurements were conducted with a Nova 4000e from Quantachrome. Therefore, nanoparticle samples were outgassed at 350°C for 1 h. N_2_ gas adsorbed onto the particle surface at 77 K and multiple measurement points were recorded to construct the BET adsorption isotherms, from which the *SSA* values were determined. The calculations were conducted with NovaWin 11.03 software from Quantachrome. For estimation of the average spherical nanoparticle diameter *d*, *SSA* can be converted according to Equation (2), where *ρ* is the bulk density of the nanoparticle material (maghemite).

(2) $$d=\frac{6}{\rho \cdot SSA}\;$$

For ATR‐FTIR spectroscopy 50 spectra per sample were recorded on an attenuated total reflectance stage (diamond/ZnSe crystal MIRacle Single Reflection ATR from PIKE Technologies) between 4000 and 650 cm^−1^ at a resolution of 4 cm^−1^ and averaged with an IR prestige 21 from Shimadzu. H_2_O and CO_2_ vibration bands were removed with the internal atmospheric correction via IRsolution 1.60 software from Shimadzu. Transmittance data were normalized between 4000 and 650 cm^−1^ for better comparability of band intensities. The quantification of the binary SAM shares was performed according to a previously published approach [47].

TGAs were run with a TG 209 F1 Libra from Netzsch. The temperature was continuously increased from room temperature to 1000°C at 30°C min^−1^ while being exposed to a gas flow of 10 mL min^−1^ of O_2_ and 40 mL min^−1^ of N_2_. The obtained curves were smoothed and derived with Proteus Thermal Analysis 8.0.3 software from Netzsch. The TGA measurements were normalized to 150°C. By determining the relative mass loss between 150 and 700°C in percent *wl*, *GD*_PA_ can be estimated according to Equation (3) (assuming that any mass loss comes from the SAM). *N*_A_ is Avogadro's constant and *MW*_PA_ the molar mass of the respective phosphonic acid derivative subtracted by the mass of the phosphonic acid group. For binary SAMs, *MW_PA_* was approximated. Therefore, the average molar mass of both derivatives was weighted by the respective surface shares determined via ATR‐FTIR spectroscopy.

(3) $$GD_{\mathrm{PA}}=\frac{wl}{100-wl}\cdot \frac{N_{A}}{MW_{\mathrm{PA}}\cdot SSA}$$

Hydrodynamic agglomerate sizes (Z‐averages) and ζ‐potentials were estimated via DLS and ELS at pH values between 3 and 11 in water with a Zetasizer Nano ZS from Malvern Instruments (633 nm laser with 173° non‐invasive backscatter detection; software version 7.13). For these measurements, the SPIONs were redispersed in DI water immediately after functionalization. Unfunctionalized SPIONs were washed with methanol and then dispersed in DI water. For each pH value, a reservoir of DI water was titrated via 0.25 M hydrochloric acid and sodium hydroxide solutions. Aliquots of 10 mL from the reservoir were spiked with 200–300 µL of the SPION dispersion. The final pH value was measured after spiking. Measurements were conducted in DTS1070 cells from Malvern Instruments. The Z‐average was measured three times per sample and averaged. The ζ‐potentials were measured five times per sample and averaged. These were fitted with a Boltzmann sigmoidal function in Origin 2024b with standard settings to estimate the respective values at pH 7 as well as the isoelectric points.

### 7.4 Magnetic Estrogen Removal

For preparation of the individual cleaning experiments, the respective estrogen was dissolved in methanol at 1 mm. This solution was diluted with DI water to obtain a 10 µm aqueous solution with 1 vol% of methanol. Another dilution to 10 nm or 1 µm was conducted using DI water with 1 vol% of methanol. Afterwards, the pH was titrated to 7 via 0.25 M hydrochloric acid and sodium hydroxide solutions. An aliquot of 7 mL of this solution was then given to 1.00 ± 0.03 mg or 10.0 ± 0.1 mg (weighing tolerances) of functionalized SPIONs, which were pre‐dispersed in 50 µL of methanol. This dispersion was vortex‐mixed at 500 rpm for 10 min with a ZX4 from VELP Scientifica. Finally, the SPIONs carrying the estrogens were collected with NdFeB, N45 magnets for 30 min against gravity (see Figures S9, S10, and S13). For preparation of the competitive cleaning experiments, equal volumes of 10 µm estrogen solutions in DI water with 1 vol% of methanol were mixed and diluted as before to obtain solutions containing 10 nm or 1 µm of each derivative. The rest of the procedure remained the same as described before. For the river water experiments, the 10 µm solution containing all estrogens was instead diluted with sampled river water spiked with 1 vol% of methanol. The natural pH was determined at 7.79 and 8.18 and not titrated. The individual cleaning of estrogens from 10 nm solutions was done in triplicates (see Figure 2a). The competitive cleaning from 10 nm DI water‐based and from river water‐based solutions were carried out in sextuplicates (see Figure 2b). The treatment with increased masses of SPIONs was done in triplicates (Figure 2c), just as the extraction of E_2_ from 1 µm starting concentration as reference for SANS and ss‐NMR spectroscopy analysis. All experiments quantified via UHPLC‐DAD were conducted in triplicates, too (Figure S16).

### 7.5 Quantification of Magnetically Removed Estrogens

1.5 mL aliquots of the treated aqueous solutions were centrifuged for 30 min at 15 000 rpm and 20°C in a Mikro 200R from Hettich. Untreated references were taken from the same initial estrogen‐spiked solutions and treated similarly to the actual samples. Treated samples and corresponding references were analyzed in the same batch via a standard‐free UHPLC‐MS^2^ method in sMRM mode. The chromatographic separation method is based on Nováková et al. [57]. Aliquots of 100 µL were injected and eluted at 0.25 mL min^−1^ by a Dionex UltiMate 3000 UHPLC system from Thermo Fisher Scientific. The estrogens were separated isocratically by a mobile phase composed of 1:1:1 ultrapure water, methanol, and acetonitrile. As columns a reversed‐phase Acquity UPLC BEH C_18_ column (150 mm length, 2.1 mm diameter and 1.7 µm particle size of filling) protected by a similar 5 mm long VanGuard pre‐column both from Waters were used (see chromatograms in Figures S11 and S14). The column and sampler temperatures were kept at 25°C. The flow was fed into a QTrap 4000 mass spectrometer with an electrospray ionization source from Sciex. Polarity of ionization was negative, the source temperature was set to 650°C, ion spray voltage to ‐4200 V, the curtain gas to 20 psig, nebulizer gas to 60 psig, heating gas to 75 psig and collision cell exit potential to ‐15 V. The sMRM detection window was set to 200 s around the typical retention times. For every derivative three or four product ions were filtered and one each was chosen as quantifier: 145.0 Da (E_1_), 269.2 Da (EE_2_), 183.0 Da (E_2_) and 171.0 Da (E_3_). All sMRM parameters and *m/z* ratios are summarized in Table S2. To quantify the removed amount of each estrogen, the quantifier product ion peak areas after treatment were referred to triplicate averages of the same peak areas from untreated references (see peak area‐concentration correlations in Figure S12). After every injection the column was rinsed with acetonitrile. All analyses were conducted via Analyst 1.6.2 from Sciex. Since fluctuations in the electrospray ionization [58] affect this relative quantification twice, removal amounts may be mathematically estimated below zero. These values were included in averaging but only means at least one standard deviation apart from no removal were interpreted as successful treatment. For quantification with a diode array detector (DAD, see Figure S16), 10 µL aliquots were injected and eluted at 0.25 mL min^−1^ by a Dionex UltiMate 3000RS UHPLC system from Thermo Fisher Scientific with the same mobile phase and column as described before. The column temperature was kept at 25°C. The flow was fed to a DAD recording the absorption at 200 nm. Again, the absorption peak areas after treatment were referred to triplicate averages of the same peak areas from untreated references (see peak area‐concentration correlations in Figure S15). After every injection the column was rinsed with acetonitrile, too. All analyses were performed via Chromeleon 7.2.10 from Thermo Fisher Scientific.

### 7.6 Estrogen–SAM Interface Characterization

SANS data were acquired with the universal high dynamic range small angle diffractometer D22 at the Institut Laue‐Langevin in Grenoble (https://doi.ill.fr/10.5291/ILL‐DATA.9‐12‐698) [59]. To mimic a typical magnetic cleaning experiment, E_2_ was dissolved in acetonitrile and diluted with degassed D_2_O to obtain a 1 µm heavy aqueous solution with 1 vol% of acetonitrile. 7 mL of this solution were given to 1.00 ± 0.03 mg of PAC_3_Imi+/PAC_18_@SPIONs pre‐dispersed in 50 µL of acetonitrile. This dispersion was vortex‐mixed at 500 rpm for 10 min with a ZX4 from VELP Scientifica. Subsequently, an aliquot was filled into a 404‐QS quartz cuvette with 5 mm path length from Hellma. For reference, the same procedure was followed but without any dissolved E_2_. The cuvettes were put in a tumbling rack and continuously rotated during the measurements. A circular neutron beam of 12 mm diameter with wavelength of 6 Å was directed onto the samples. The scattered neutrons were detected at a rear detector distance of 4 m (collimation 4 m). The additional front detector was positioned at 1.4 m. This enabled a *q* detection range between 0.014 and 0.610 Å^−1^. Therefore, two ^3^He detectors consisting of 128 (for the rear detector) and 96 (for the front detector) vertically aligned Reuter‐Stokes tubes all with a diameter of 8 mm (pixel size of 8 mm x 8 mm) were used. The scattering images were corrected to the transmission of the direct beam, scaled to absolute intensity, and azimuthally averaged using the GRASP software [60]. Empty cell and solvent scattering were subtracted from the obtained scattering curves. The records were analyzed and modeled using the SasView 5.0.6 software package [61]. *P*_core_, *P*_core‐shell_ and *P*_core‐shell‐shell_ were derived from the included “onion” form factor model. The SLD profiles are illustrated in Figure S17. For fitting, the DREAM algorithm with 10^6^ samples for each initial fit was used. Scales and background were additionally optimized with 10^5^ samples.

For ss‐NMR spectroscopy, functionalized TiON samples without E_2_ were dried at 80°C for 2 h. Afterwards, the atmosphere was replaced with nitrogen for storage. To mimic the magnetic cleaning procedure, PAC_3_Imi+/PAC_18_@TiONs were pre‐dispersed in methanol and treated with aqueous 1 µm E_2_ solution (pH 7, 1 vol% of methanol) at the same mass‐to‐volume ratio as in the magnetic cleaning experiments. The dispersions were vortex‐mixed at 200 rpm for 10 min with a ZX4 from VELP Scientifica followed by sedimentation via centrifugation at 12 000 rpm for 30 min with a Multifuge X1R from Heraeus since no magnetic collection was possible. The particles were dried at 60°C over‐night and at 80°C for 2 h prior to storage in nitrogen atmosphere. All spectra were recorded with an 800 MHz (18.8 T) Ascend NMR spectrometer from Bruker. MAS was performed in 1.3 mm double‐resonance probes at 50 kHz spinning rate. The ^1^H high‐field spectra were recorded at 18.8 T with a frequency of 800.13 MHz. For every spectrum, 16 scans were collected at a delay time of 3 s. All spectra were referenced to tetramethylsilane using adamantane as a secondary reference resonating at 1.85 ppm. Every spectrum was background‐corrected and mass‐normalized. Spectra deconvolution was carried out with Dmfit #20230120 software [62]. The ^1^H‐^1^H DQ BABA experiments were performed with similar rotors and spinning rates. 128 points were collected in the indirect dimension with 16 scans each (96 for the binary SAM coated TiONs before and after E_2_ adsorption). All data were evaluated with TopSpin 4.1.3 software from Bruker [63].

All methods for analysis of SAMs on flat substrates are described in the Supporting Information.

### 7.7 MD Simulation

The MD simulations were carried out with the LAMMPS package using a time step of 1 fs [64]. Constant temperature and pressure were maintained by the Nosé‐Hoover algorithm using relaxation constants of 0.5 and 1.0 ps, respectively [65]. The atomic interactions were described by molecular mechanics force‐fields as described in the Supporting Information using a cut‐off delimiter of 1.2 nm. To mimic long‐range electrostatics, damped‐shifted force Coulomb potentials were applied [66].

A spherical SPION nanoparticle model with a radius 6.3 nm was prepared by melting a 10 × 10 × 10 nm maghemite block and quenching from 2000 K to room temperature. After equidistant placement of PAC_3_Imi+ (with Br^−^) and PAC_18_ at 20:80 ratio (1.66 molecules per nm^2^) on the nanoparticle, the functionalized SPION was first equilibrated in vacuum and then solvated in a melt of E_2_. From this virtual model, we randomly chose ten E_2_ molecules to obtain diverse and unbiased starting configurations. Stripping off all other hormone molecules, the now loaded SPION was immersed in water and allowed to relax. Upon 5 ns equilibration at 300 K and 1 atm, statistics of the binding poses were collected from additional 30 ns runs. For the sake of comparability, the same starting poses were used for all E_1_, EE_2_, and E_3_, too. To benchmark statistical quality, three independent runs were performed for each system (featuring E_1_, E_2_, EE_2_, and E_3_, respectively).

Coordination numbers were assessed from radial distribution functions using the minimum between first and second coordination peaks as distance delimiters (see Figures S37–S44). To distinguish between the binding motifs, we analyzed the coordination environment of the hormone molecules, namely the hydrophobically coordinated water oxygens within 4 Å around each pollutant, as well as hydrogen bonding partners within 2.5 Å of either oxygen or hydroxyl hydrogen. For E_1_, E_2_, EE_2_, and E_3_ we identified type III coordination as molecules with less than 5 water molecules in hydrophobic contact and less than 4, 6, 4 and 6 hydrogen bonds to water, respectively, as well as all poses with less than 10 hydrophobic contacts, but with at least one hydrogen bond to O^2−^. We then calculated mean values for the number of interaction partners for each derivative in either type I/II or type III coordination (see Table S8).

Insight into the thermodynamics of estrogen association to the SAM interface was generated via Boltzmann‐derived energy calculations at 750 K, subtracting a correction term for the entropy of dilution. Thus, we calculated the difference in chemical potential Δ*µ* between bound and hydrated pollutant molecule states. For calculation of ∆*µ*, we determined the accessible volumes for bound and freely dispersed estrogen molecules in each simulation, respectively (see Equation S5). Therefore, we used a distance‐dependent cutoff delimiter *d*  =  2.5 Å around the mean diameter of the functionalized SPION, whereas the remaining space of the simulation system was considered as the volume of the water phase. The hydrated molecules from the high‐temperature simulations were also characterized in short simulations at room temperature to compare their coordination environment to the type I/II and type III associated molecules. For the high‐temperature runs performed in vacuum, we used the solvated systems as starting points and fixed the simulation cell to constant volume before removing the water molecules.

A more detailed methods part of the MD simulation can be found in the Supporting Information.

## Author Contributions

Conceptualization, L.M. and M.Ha.; Methodology, L.M., P.M., D.V., W.M., N.L., T.M.S., I.K., M.Hu., W.C., C.C., L.Ru., H.G., J.B., K.V., H.G.S., S.D., E.B., R.S., D.Z., and M. Ha.; Formal analysis, L.M., P.M., D.V., W.M., N.L., I.K., M.Hu., W.C., C.C., L.Ru., J.V., K.V., and E.B.; Investigation, L.M., P.M., D.V., W.M., N.L., T.M.S., J.F., I.K., M.Hu., W.C., C.C., L.Ru., J.V., L.Ro., K.V., and H.G.S.; Resources, L.M., J.B., G.G., K.V., H.G.S., E.S., S.D., M.P., E.B., R.S., D.Z., and M.H a.; Writing – original draft, L.M.; writing – review & editing, all authors; Visualization, L.M., P.M., D.V.; Supervision, G.G., K.V., H.G.S., E.S., S.D., M.P., E.B., R.S., D.Z., and M.Ha.; Project administration, L.M. and M.Ha.; Funding acquisition, L.M., W.M., M.Hu., W.C., J.V., H.G.S., and M.Ha.

## Disclosure

No materials from other sources were reproduced.

## Ethics Statement

No ethics‐relevant experiments were performed in this work. Therefore, no ethical approval is required.

## Consent

No clinical trials were conducted in this work.

## Conflicts of Interest

The authors declare no conflicts of interest.

## Data and Code Availability

All data supporting this study are openly available at the Figshare data repository (https://doi.org/10.6084/m9.figshare.31400514) [67]. There, also code used for XRR and GIWAXS measurements is available.

## Supporting information




**Supporting File 1**: advs77321‐sup‐0001‐SuppMat.docx.

**Supporting File 2**: advs77321‐sup‐0002‐Video1.mp4.

**Supporting File 3**: advs77321‐sup‐0003‐Video2.mp4.

**Supporting File 4**: advs77321‐sup‐0004‐Video3.mp4.

**Supporting File 5**: advs77321‐sup‐0005‐Video4.mp4.

**Supporting File 6**: advs77321‐sup‐0006‐Video5.mp4.

## Data Availability

The data that support the findings of this study are openly available in Figshare at https://doi.org/10.6084/m9.figshare.31400514.
