# Supplementary material for: Magnetic Water Cleaning of Estrogens via Tight yet Dynamic Binding to Nanoparticles
Source: Adv Sci (Weinh). 2026 Sep 13:e77321. Online ahead of print. doi: 10.1002/advs.77321 (PMC13572863; doi:10.1002/advs.77321)
Supplement: Supplementary file 1 [file ADVS-9999-e77321-s005.docx]

Supplementary information

Magnetic water cleaning of estrogens via tight yet dynamic binding to nanoparticles

*Lukas Müller^1,2*^, Philip Maier^3^, Dustin Vivod^3^, Wenke Müller^4^, Nikolaj Lopatik^5^, Theresa Maria Schichtl^6^, Julia Fröhlich^1^, Irene Kraus^7^, Marten Huck^8^, Wieland Corts^8^, Clodomiro Cafolla^9^, Lukas Ruhm^10^, Henrik Gaß^1^, Johannes Voß^1^, Jiwon Byun^1^, Linda Rockmann^1^, Guido Grundmeier^10^, Kislon Voïtchovsky^9^, Hans-Georg Steinrück^8^, Erdmann Spiecker^7^, Sevim Dalabasmaz^6,11^, Monika Pischetsrieder^6,11^, Eike Brunner^5^, Ralf Schweins^4^, Dirk Zahn^3^ and Marcus Halik^1,*^*

^*^shared corresponding authors: lukas.muller@colorado.edu, marcus.halik@fau.de

^1^Organic Materials & Devices, Institute of Polymer Materials, Friedrich-Alexander-Universität Erlangen-Nürnberg, Interdisciplinary Center for Nanostructured Films (IZNF), Cauerstraße 3, 91058 Erlangen, Germany

^2^RASEI: The CU-Boulder/NLR Energy Institute, University of Colorado Boulder, 4001 Discovery Drive, Boulder, Colorado 80309, United States

^3^Computer Chemistry Center & Chair of Theoretical Chemistry, Friedrich-Alexander-Universität Erlangen-Nürnberg, Nägelsbachstraße 25, 91052 Erlangen, Germany

^4^Institut Laue-Langevin, DS/LSS, 71 Avenue des Martyrs, 38000 Grenoble, France

^5^Chair of Bioanalytical Chemistry, Faculty of Chemistry and Food Chemistry, Technische Universität Dresden, Bergstraße 66, 01062 Dresden, Germany

^6^Food Chemistry, Department of Chemistry and Pharmacy, Friedrich-Alexander-Universität Erlangen-Nürnberg, Nikolaus-Fiebiger-Straße 10, 91058 Erlangen, Germany

^7^Institute of Micro- and Nanostructure Research (IMN) & Center for Nanoanalysis and Electron Microscopy (CENEM), Friedrich-Alexander-Universität Erlangen-Nürnberg, Interdisciplinary Center for Nanostructured Films (IZNF), Cauerstraße 3, 91058 Erlangen, Germany

^8^Institute for a Sustainable Hydrogen Economy, Forschungszentrum Jülich GmbH, An der Deutschen Welle 7a, 52428 Jülich, Germany & Institute of Physical Chemistry, RWTH Aachen University, Landoltweg 2, 52074 Aachen, Germany & Chemistry Department, Universität Paderborn, Warburger Straße 100, 33098 Paderborn, Germany

^9^Physics Department, Durham University, South Road, Durham DH1 3LE, United Kingdom

^10^Technical and Macromolecular Chemistry, Universität Paderborn, Warburger Straße 100, 33098 Paderborn, Germany

^11^FAU NeW − Research Center New Bioactive Compounds, Friedrich-Alexander-Universität Erlangen-Nürnberg (FAU), Nikolaus-Fiebiger-Straße 10, 91058 Erlangen, Germany

**Table of contents**

[Comprehensive characterization of functional SPIONs	3](#_Toc222845137)

[Magnetic water cleaning quantification details, photographs of all SPION dispersions, and explanation for estimated estrogen coverages	12](#_Toc222845138)

[SANS evaluation details and support	22](#_Toc222845139)

[Characterization of TiON model system	26](#_Toc222845140)

[Reference ss-NMR spectra and deconvolution	29](#_Toc222845141)

[Comprehensive study of functional SAMs on flat AlO_x_ substrates	37](#_Toc222845142)

[MD simulation support, coordination analyses, and methodology	49](#_Toc222845143)

[References	62](#_Toc222845144)

# Comprehensive characterization of functional SPIONs

**Figure S1: Characterization of commercial SPIONs.**

**a-f**, HR-TEM micrograph (**a**), SAED pattern and radial diffractogram with theoretical peak positions (black lines) as well as indices (**b**, **c**), N_2_ gas adsorption BET isotherms with derived *SSA* values before and after dispersion in methanol (**d**),^1^ DLS-recorded Z-averages and ELS-recorded ζ-potentials at various pH values in water fitted with a Boltzmann sigmoidal function as well as ζ-potential at pH 7 with 95 % confidence interval (**e**) and a representative ATR-FTIR spectrum (**f**). The nanoparticles have an average size of 10.7 nm calculated from their *SSA* assuming sphericity, which agrees with the micrograph. They are composed of either magnetite or maghemite according to SAED, from which we conclude on maghemite due to their brown-red color. The observed diffraction peaks and the theoretically expected peaks derived from the structure factor of maghemite agree, as indexed and marked accordingly. Furthermore, the SPIONs have an isoelectric point around pH 7 and form µm-scaled agglomerates in water around this pH value. Vibrational bands around 3400, 3220, 1430, 1340, 1120, 1040 and 895 cm^-1^ can be assigned to either NH, CH or CO vibrations while the band at 1630 cm^-1^ indicates double bond CC or CN stretching vibrations. Thus, the commercial particles are likely covered with an unknown short chained amino-terminated carboxylic acid stabilizer. Due to the high binding strength of phosphonic acids, this stabilizer can be replaced upon functionalization. Data are represented as mean ± standard deviation from technical *n* = 3 (DLS) and technical *n* = 5 (ELS) combined from two independent titration series (**e**).

**Figure S2: Representative ATR-FTIR spectra of SAM-coated SPIONs.**

**a**, **b**, Transmittance ATR-FTIR spectra (**a**) and absorbance spectra segments (**b**) of SAM-coated SPIONs with references. Weak bands around 3150 and 3080 cm^-1^ (NH or CH in imidazolium ring), around 2960, 2925, 2900 and 2850 cm^-1^ (CH_2_ and CH_3_), around 1560 cm^-1^ (CN in imidazolium ring) and stronger vibrations between 1250-850 cm^‑1^ (PO) confirm the functionalization with PAC_3_Imi+. For PAC_18_@SPIONs, strong bands around 2955, 2920, 2850 and 1460 cm^-1^ (aliphatic CH_2_ and CH_3_) as well as a convoluted band from 1160-835 cm^-1^ (PO) confirm the functionalization. For PAC_3_Imi+/PAC_18_@SPIONs, the vibrational bands of both molecules are found, however, PAC_18_ strongly dominates. By referring aliphatic, asymmetrical CH_2_ $\upsilon\left( \mathrm{CH}_{2}^{\mathrm{asym}} \right)$ to aliphatic, asymmetrical CH_3_ $\upsilon\left( \mathrm{CH}_{3}^{\mathrm{asym}} \right)$ vibrational absorbance intensities (**b**), the surface share of both molecules in the binary SAM can be estimated.^2^ In PAC_3_Imi+/PAC_18_@SPIONs about 15 % of the binary SAM are PAC_3_Imi+ and about 85 % PAC_18_ (±4 %) as determined from *n* = 2. The SPION systems spectra in **a** are stretched by a factor of two relative to the molecule references.

**Figure S3: TGA and derived GD_PA_ of SAM-coated SPIONs.**

**a**, **b**, TGA including DTG (**a**) and calculated *GD_PA_* (**b**) of SAM-coated SPIONs with unfunctionalized SPIONs as reference. All systems show greater mass losses between 150 and 700 °C than unfunctionalized SPIONs indicating a substantial organic coverage in all cases (PAC_3_Imi+@SPIONs: 4.5 ± 0.3 %, PAC_3_Imi+/PAC_18_@SPIONs: 7.9 ± 0.2 %, PAC_18_@SPIONs: 9.7 ± 0.4 %). These mass losses are converted to *GD_PA_* estimates assuming the full SSA is available and all stabilizer replaced (PAC_3_Imi+@SPIONs: 1.2 ± 0.1 nm^-2^, PAC_3_Imi+/PAC_18_@SPIONs: 1.8 ± 0.1 nm^-2^, PAC_18_@SPIONs: 2.4 ± 0.2 nm^-2^). Thus, the positively charged PAC_3_Imi+ SAM packs about half as dense as PAC_18_, while the binary SAM is in between. The mass losses between 700 and 900 °C may be attributed to iron oxide phase changes. Data are shown as mean ± standard deviation from *n* = 3 in **a** and from *n* = 12 for PAC_3_Imi+@SPIONs, *n* = 7 for PAC_18_@SPIONs and *n* = 3 for PAC_3_Imi+/PAC_18_@SPIONs in **b**.

**Figure S4: DLS-recorded Z-averages and ELS-recorded ζ-potentials of SAM-coated SPIONs at various pH values in water.**

For PAC_3_Imi+@SPIONs µm-scaled agglomerates are found around pH 7, whereas PAC_18_@SPIONs do not follow a specific trend with pH. This points to a significant electrostatic stabilization contribution in the first case. PAC_3_Imi+/PAC_18_@SPIONs show an intermediate behavior when comparing the DLS-derived Z-averages. If compared to the photographs in Figure S10 and Figure S13, it becomes clear that the given DLS-derived sizes are non-representative for the actual dispersion quality (especially for PAC_18_@SPIONs, which are dispersed worst) due to segregating agglomerates, which is why they are not further discussed. The given ζ-potentials (at pH 7 with a 95 % confidence interval derived from fitted Boltzmann sigmoidal functions) show that PAC_18_@SPIONs get more negative compared to the unfunctionalized SPIONs, while PAC_3_Imi+@SPIONs remain similar. The ζ-potential of PAC_3_Imi+/PAC_18_@SPIONs is close to the latter, despite the majority of the binary SAM being PAC_18_. Likely the phosphonic acid anchors are negatively charged, which is compensated by the positively charged imidazolium in PAC_3_Imi+ resulting in a zwitterionic state on the SPIONs, thus, not strongly affecting the overall ζ-potential while simultaneously increasing water dispersibility significantly. Data are represented as mean ± standard deviation from technical *n* = 3 (DLS) and technical *n* = 5 (ELS) combined from mostly two (three for PAC_3_Imi+@SPIONs) independent titration series.

**Table S1: Conversion of solution to surface ratio of PAC_3_Imi+ and PAC_18_ in co-self-assembled binary SAMs on SPIONs.**

The surface shares were estimated via ATR-FTIR spectroscopy (see Figure S5) according to a previously published procedure.^2^ PAC_18_ is found on the SPION surface in stoichiometric excess. The nomenclature in the right column is used in the upcoming figures. Data was estimated from *n* = 2.

| Solution ratio between PAC_3_Imi+ and PAC_18_ | Share on surface  PAC_3_Imi+  [%] | Share on surface  PAC_18_  [%] | Standard deviation  [%] | Nomenclature by surface ratio |
| --- | --- | --- | --- | --- |
| 95/5 | 79 | 21 | 4 | $\sim$ 80/20 |
| 90/10 | 48 | 52 | 5 | $\sim$ 50/50 |
| 80/20 | 22 | 78 | 4 | $\sim$ 20/80 |
| 50/50 | 7 | 93 | 7 | $\sim$ 10/90 |

**Figure S5: Representative ATR-FTIR spectra of simultaneously co-self-assembled PAC_3_Imi+&PAC_18_@SPIONs.**

**a**, **b**, Absorbance ATR-FTIR spectra segments (**a**) and derived surface shares depending on the solution shares of PAC_18_ as given in Table S1 (**b**). **c**, Transmittance ATR-FTIR spectra of the respective samples including unitary SAM references. By referring aliphatic, asymmetrical CH_2_ $\upsilon\left( \mathrm{CH}_{2}^{\mathrm{asym}} \right)$ to aliphatic, asymmetrical CH_3_ $\upsilon\left( \mathrm{CH}_{3}^{\mathrm{asym}} \right)$ vibrational absorbance intensities, the surface share of both molecules in the binary SAM can be estimated.^2^ Especially, the aliphatic vibrational bands around 2955, 2920, 2850 and 1460 cm^-1^ (CH_2_ and CH_3_) and the stronger convoluted band from 1160-835 cm^-1^ (PO) indicate a stoichiometric excess of PAC_18_. Data in **b** are represented as mean ± standard deviation estimated from *n* = 2.

**Figure S6: TGA and derived GD_PA_ of simultaneously co-self-assembled PAC_3_Imi+&PAC_18_@SPIONs.**

**a**, **b**, TGA including DTG (**a**) and calculated *GD_PA_* (**b**) of simultaneously co-self-assembled PAC_3_Imi+&PAC_18_@SPIONs in various ratios with unfunctionalized SPIONs as reference. The *GD_PA_* values were calculated taking into account the respective ATR-FTIR spectroscopy determined surface shares. Overall, these values stay similar to the one of PAC_3_Imi+ until increasing to the one of PAC_18_ between $\sim$ 20/80 and $\sim$ 10/90 surface shares. Data are shown as mean ± standard deviation mostly from *n* = 3 ($\sim$ 80/20, $\sim$ 50/50 and $\sim$ 20/80 PAC_3_Imi+&PAC_18_@SPIONs from *n* = 2) in **a** as well as from *n* = 12 for PAC_3_Imi+@SPIONs, *n* = 7 for PAC_18_@SPIONs, *n* = 3 for $\sim$ 10/90 PAC_3_Imi+&PAC_18_@SPIONs and *n* = 2 for the rest in **b**.

**Figure S7: DLS-recorded Z-averages and ELS-recorded ζ-potentials of simultaneously co-self-assembled PAC_3_Imi+&PAC_18_@SPIONs at various pH values in water.**

The previously discussed µm-scaled agglomeration around pH 7 is observable until $\sim$ 20/80 surface share, while the ζ-potentials at pH 7 with 95 % confidence intervals derived from fitted Boltzmann sigmoidal functions remain close to the one of PAC_3_Imi+@SPIONs for all binary SAMs. Data are represented as mean ± standard deviation from technical *n* = 3 (DLS) and technical *n* = 5 (ELS) combined from mostly two (three for PAC_3_Imi+@SPIONs) independent titration series.

**Figure S8: ELS-derived isoelectric points of simultaneously co-self-assembled PAC_3_Imi+&PAC_18_@SPIONs in water.**

The isoelectric points of all binary SAM systems are similar to the one of PAC_3_Imi+@SPIONs, thus, showing the dominating role of PAC_3_Imi+ on the colloidal properties. The data are given with 95 % confidence intervals derived from the Boltzmann sigmoidal function fits in Figure S7.

# Magnetic water cleaning quantification details, photographs of all SPION dispersions, and explanation for estimated estrogen coverages

**Figure S9: Illustration of the magnetic water cleaning process and quantification.**

Functional SPIONs and estrogen-spiked solution are combined (i), vortex-mixed for 10 min (ii) and the estrogen-laden SPIONs magnetically collected for 30 min against gravity (iii). The removed estrogen amounts were quantified by referring peak areas of treated solutions to peak areas of untreated reference solutions via UHPLC-MS^2^ in sMRM mode (iv).

**Figure S10: Photographs of functional SPION dispersions similar to cleaning experiments before and after magnetic collection.**

At the water cleaning experiment conditions (1.00 ± 0.03 mg of functional SPIONs pre-dispersed with 50 µL of methanol in 7 mL of DI water with 1 vol% methanol titrated to pH 7), PAC_3_Imi+@SPIONs and PAC_3_Imi+/PAC_18_@SPIONs are dispersed better than PAC_18_@SPIONs, which mostly float on the surface (i-iv). Regardless of SAM, all SPIONs can be visually removed from the water phase by applying a magnet for 30 min (v-ix).

**Table S2: sMRM mode parameters and estrogen-specific precursor as well as product ions.**

Product ions with *m/z* ratios of 145.0 Da (E_1_), 269.2 Da (EE_2_), 183.0 Da (E_2_) and 171.0 Da (E_3_) were used as quantifiers, while the rest served as qualifiers. The individual declustering and entrance potentials as well as collision energies were determined from parameter variation for each estrogen.

| Analyte/  estrogen | Molecular  weight  [g mol^-1^] | Precursor  ion  [Da] | Declustering  potential  [V] | Entrance  potential  [V] | Collision  energies  [eV] | Product  ions  [Da] |
| --- | --- | --- | --- | --- | --- | --- |
| E_1_ | 270.4 | 269.1 | -130 | -9 | -80 | 143.0 |
|  |  |  |  |  | -55 | 145.0 |
|  |  |  |  |  | -50 | 159.0 |
|  |  |  |  |  | -50 | 183.1 |
| EE_2_ | 296.4 | 295.2 | -100 | -14 | -55 | 145.0 |
|  |  |  |  |  | -55 | 159.0 |
|  |  |  |  |  | -55 | 183.0 |
|  |  |  |  |  | -45 | 269.2 |
| E_2_ | 272.4 | 271.2 | -90 | -12 | -55 | 145.1 |
|  |  |  |  |  | -55 | 183.0 |
|  |  |  |  |  | -55 | 239.1 |
| E_3_ | 288.4 | 287.2 | -80 | -11 | -70 | 143.0 |
|  |  |  |  |  | -55 | 159.0 |
|  |  |  |  |  | -50 | 171.0 |
|  |  |  |  |  | -50 | 183.0 |

**Figure S11: Representative chromatogram of estrogens at 10 nM each measured via UHPLC-MS^2^ in sMRM mode.**

The chromatogram is combined from the respective sMRM recordings of the quantifier ions given in Table S2. E_3_ shows the by far shortest retention time when passing through the reversed-phase column, while E_2_ is eluted before EE_2_ and E_1_. Accordingly, the hydrophobicity of the estrogen derivatives is ordered as E_1_>EE_2_>E_2_>>E_3_.

**Figure S12: Signal-concentration correlations between 1 and 10 nM for estrogens determined via UHPLC-MS^2^ in sMRM mode.**

**a**-**d**, Peak areas (from quantifier ions given in Table S2) are found in reasonable linear relation to concentration for all estrogen derivatives in the relevant concentration regime. Data are represented as mean ± standard deviation from *n* = 3.

**Figure S13: Photographs of higher-concentrated functional SPION dispersions before and after magnetic collection.**

The photographs show 10.0 ± 0.1 mg of PAC_18_@SPIONs and PAC_3_Imi+/PAC_18_@SPIONs pre-dispersed with 50 µL methanol in 7 mL of DI water with 1 vol% methanol titrated to pH 7 (i-ii). With this higher mass of SPIONs, it becomes even clearer that the binary SAM coated SPIONs in ii) are much better dispersed in the water phase than PAC_18_@SPIONs in i). Regardless of SAM, both SPION systems can be visually removed from the water phase by applying a magnet for 30 min (iii-v).

*Explanation on estimated estrogen coverages*

The estrogen coverage on the functional SPIONs can be exemplarily estimated for treatment of 7 mL of mixtures containing 280 pmol of estrogens (10 nM) with 1.00 ± 0.03 mg of PAC_18_@SPIONs as given in Figure 2, b. Overall, 212.6 ± 11.2 pmol of estrogens were removed, *i.e.*, adsorbed on the SPIONs. Since the actual estrogen-available surface area is experimentally not accessible, two extreme scenarios of available *SSA* can be distinguished. Firstly, the reasonable maximum for an estrogen-available *SSA* is the BET-derived value of 115.1 m^2^ g^-1^. On the other hand, µm-scaled agglomerates were found in water. If spherical, however, completely non-porous 1 µm agglomerates are assumed, a *SSA* of 1.2 m^2^ g^-1^ would be available for estrogen adsorption. These values translate to an estimation interval between 0.001 and 0.1 of estrogens per nm^2^ of SPION surface for the applied mass. If compared to monolayer *GD_PA_* values, these small estrogen coverages (that are also not limited to form a monolayer only) show non-saturation of the surface at the investigated pollutant concentrations and adsorbent masses. Similarly, both scenarios can be applied to estimate the coverage of E_2_ in the SANS experiments, when applying 1.00 ± 0.03 mg of PAC_3_Imi+/PAC_18_@SPIONs to 7 mL aqueous solution containing 7 nmol (1 µM) of E_2_. Here, 3.90 ± 0.28 nmol of E_2_ were adsorbed, which translates with the previously discussed available *SSA* values to a coverage estimation between about 0.02 and 1.91 E_2_ molecules per nm^2^ of SPION surface for the applied mass. While showing greater coverage, this still suggests below monolayer adsorption. Due to the significant influence on the scattering pattern, we believe that the real value is closer to 1.91 molecules per nm^2^.

**Figure S14: Representative chromatograms of estrogens at 1 µM each measured via UHPLC-DAD.**

The UHPLC-DAD chromatogram displays the absorbance at 200 nm. The dashed box shows a zoom-in. The strong drop in absorbance around 1.7 min is due to elution of the injection matrix.

**Figure S15: Signal-concentration correlations between 0.1 and 10 µM for estrogens determined via UHPLC-DAD.**

**a**-**d**, Peak areas (measured at 200 nm absorbances) are found in reasonable linear relation to concentration for all estrogen derivatives in the relevant concentration regime. Data in **c** are represented as mean ± standard deviation from *n* = 3.

**Figure S16: Magnetic water cleaning performances of functional SPIONs at higher starting concentrations.**

**a**, Removed amounts are given from treatment of aqueous solutions containing 7 nmol of each estrogen (7 mL à 1 µM each at pH 7) with 1.00 ± 0.03 mg of functional SPIONs determined via UHPLC-DAD. **b**, Similar solutions as in **a** were also treated with 1.00 ± 0.03 mg of different compositions of simultaneously co-self-assembled PAC_3_Imi+&PAC_18_@SPIONs and removal was quantified with UHPLC-DAD. The treatment of the 100x higher concentrated solutions in **a** (compared to Figure 2) still yields considerable cleaning results with PAC_18_@SPIONs and PAC_3_Imi+/PAC_18_@SPIONs confirming non-saturated adsorption when treating 10 nM samples. Additionally, the dependency in **b** underlines the PAC_18_-driven interaction of estrogens with the binary SAM since the removed amounts increases in correlation with the PAC_18_ surface share. If the determined removal amounts are not at least one standard deviation apart from no removal, the data are marked with an asterisk (*****) and interpreted as no significant removal. Data are given as mean ± standard deviation from *n* = 3.

# SANS evaluation details and support

**Figure S17: Illustration of form factor model parameters and SLD profiles.**

For the form factor models *P_core-shell_* (i) and *P_core-shell-shell_* (ii) different SLD profiles were modelled representing two structure reductionisms of the PAC_3_Imi+/PAC_18_@SPIONs system. The fitted and fixed values are summarized in Table S3. For *P_core_* a simple profile only containing *SLD_core_* and *SLD_solvent_* was employed.

**Figure S18: Recorded and modeled SANS data for PAC_3_Imi+/PAC_18_@SPIONs without and with adsorbed E_2_ in heavy aqueous dispersion.**

**a**, **b**, Recorded and modelled SANS data from dispersions of 1.00 ± 0.03 mg of PAC_3_Imi+/PAC_18_@SPIONs in 7 mL of D_2_O with 1.7 vol% acetonitrile (**a**) and 1 µM of E_2_ additionally (**b**). The data was fitted according to Equation 1 with three form factor models: A spherical nanoparticle (*P_core_*), a spherical core-shell nanoparticle (*P_core-shell_*) and a spherical core-shell-shell nanoparticle structure (*P_core-shell-shell_*). While the overall trend can be represented with *P_core_*, the scattering between 0.02 and 0.2 Å^‑1^ is represented better with *P_core-shell_* in both cases suggesting a significant scattering contribution originating from the organic shell (even though $\chi_{\mathrm{red}}^{2}$ with *P_core_* is closer to 1 in **b**, it does not capture the intensity oscillations around 0.1 Å^‑1^). With *P_core-shell-shell_* adequate modelling is only possible with adsorbed E_2_, which underlines a double shell-like adsorption layer. In **a** this more complex form factor model does not yield an adequate result due to enforced variable values assessing its double shell character. Overall, *P_core-shell_* is suited best for discussing the data as it models both scenarios adequately. The given error bars account for *q* smearing and intensity measurement uncertainty, which both were accounted for in data modeling.

**Table S3: Modelling parameter summary and derivation for SANS evaluation.**

The underlying phosphonic acid chain lengths were estimated after structural relaxation via Chem3D version 18.1.0.535 from Perkin Elmer. *r_core_* was taken from the BET-derived average SPION diameter. *SLD_core_* corresponds to the SLD of Fe_2_O_3_ and *SLD_out_* = *SLD_out,2_* = *SLD_solvent_* to the SLD of D_2_O. *SLD_in_* for PAC_3_Imi+/PAC_18_@SPIONs was determined from a preliminary fit. *SLD_in_* with adsorbed E_2_ was chosen as average of the SLD of the binary SAM *SLD_SAM_* (0.24 $\cdot$ 10^-6^ Å^-2^ as 0.17:0.83 weighted average of *SLD_PAC3Imi+_*:*SLD_PAC18_*) and this value corrected by the content of D_2_O in the SAM at 7 Å away from the surface estimated via MD simulation (5.49 $\cdot$ 10^-6^ Å^-2^ as 0.85:0.15 weighted average of *SLD_solvent_*:*SLD_SAM_*). *d_shell_* has a lower limit of 3 Å (to avoid converging into *P_core_*) and an upper limit of 32.6 Å, which equals the sum of the chain length of PAC_18_ (21.7 Å) and the length of an E_2_ molecule (10.9 Å). *SLD_in,1_* is equal to *SLD_SAM_* and *SLD_out,1_* = *SLD_in,2_* are equal to this value corrected by the simulated D_2_O content. *d_shell,1_* is fixed to the chain length of PAC_3_Imi+ at 7 Å. Lastly, *d_shell,2_* is limited to 25.6 Å, which is the sum of the PAC_18_ chain length and the length of an E_2_ molecule subtracted by the chain length of PAC_3_Imi+, which was already accounted for in the inner shell. The necessity of a higher *SLD_in_* value for better fit quality in modelling the organic shell without E_2_ may be interpreted as more D_2_O in the shell as confirmed by MD simulation in **Figure S19**. The fitted values are given with 95 % confidence intervals.

| Model, parameters and limits | PAC_3_Imi+/PAC_18_@SPIONs | PAC_3_Imi+/PAC_18_@SPIONs + E_2_ |
| --- | --- | --- |
| $\boldsymbol{A}\boldsymbol{\cdot}\boldsymbol{q}^{\boldsymbol{-}\boldsymbol{4}}\boldsymbol{+}\boldsymbol{B}\boldsymbol{\cdot}\boldsymbol{P}_{\boldsymbol{core}}\boldsymbol{+}\boldsymbol{bkg}$ | $\chi_{\mathrm{red}}^{2}$ = 1.64 | $\chi_{\mathrm{red}}^{2}$ = 1.04 |
| *A* $\boldsymbol{\in}$ [$\boldsymbol{-\infty}$;$\boldsymbol{\infty}$] | (2.75 ± 0.23) $\cdot$ 10^-11^ | (7.22 ± 0.23) $\cdot$ 10^-11^ |
| *B* $\boldsymbol{\in}$ [$\boldsymbol{-\infty}$;$\boldsymbol{\infty}$] | (1.06 ± 0.08) $\cdot$ 10^-4^ | (6.97 ± 0.79) $\cdot$ 10^-5^ |
| *r_core_* (fixed) [Å] | 53.65 | 53.65 |
| *SLD_core_* (fixed) [Å^-2^] | 6.66 $\cdot$ 10^-6^ | 6.66 $\cdot$ 10^-6^ |
| *SLD_solvent_* (fixed) [Å^-2^] | 6.39 $\cdot$ 10^-6^ | 6.39 $\cdot$ 10^-6^ |
| *bkg* $\boldsymbol{\in}$ [$\boldsymbol{-\infty}$;$\boldsymbol{\infty}$] | (2.32 ± 0.03) $\cdot$ 10^-4^ | (2.14 ± 0.03) $\cdot$ 10^-4^ |
| $\boldsymbol{A}\boldsymbol{\cdot}\boldsymbol{q}^{\boldsymbol{-}\boldsymbol{4}}\boldsymbol{+}\boldsymbol{B}\boldsymbol{\cdot}\boldsymbol{P}_{\boldsymbol{core}\boldsymbol{-}\boldsymbol{shell}}\boldsymbol{+}\boldsymbol{bkg}$ | $\chi_{\mathrm{red}}^{2}$ = 1.31 | $\chi_{\mathrm{red}}^{2}$ = 1.30 |
| *A* $\boldsymbol{\in}$ [$\boldsymbol{-\infty}$;$\boldsymbol{\infty}$] | (3.33 ± 0.21) $\cdot$ 10^-11^ | (6.91 ± 0.28) $\cdot$ 10^-11^ |
| *B* $\boldsymbol{\in}$ [$\boldsymbol{-\infty}$;$\boldsymbol{\infty}$] | (3.75 ± 0.26) $\cdot$ 10^-4^ | (5.94 ± 0.83) $\cdot$ 10^-6^ |
| *r_core_* (fixed) [Å] | 53.65 | 53.65 |
| *SLD_core_* (fixed) [Å^-2^] | 6.66 $\cdot$ 10^-6^ | 6.66 $\cdot$ 10^-6^ |
| *SLD_in_* (fixed) [Å^-2^] | 5.70 $\cdot$ 10^-6^ | 2.87 $\cdot$ 10^-6^ |
| *SLD_out_* (fixed) [Å^-2^] | 6.39 $\cdot$ 10^-6^ | 6.39 $\cdot$ 10^-6^ |
| *SLD_solvent_* (fixed) [Å^-2^] | 6.39 $\cdot$ 10^-6^ | 6.39 $\cdot$ 10^-6^ |
| *d_shell_* $\boldsymbol{\in}$ [3.00;32.60] [Å] | 6.90 ± 2.44 | 17.65 ± 6.32 |
| *bkg* $\boldsymbol{\in}$ [$\boldsymbol{-\infty}$;$\boldsymbol{\infty}$] | (2.25 ± 0.03) $\cdot$ 10^-4^ | (2.14 ± 0.03) $\cdot$ 10^-4^ |
| $\boldsymbol{A}\boldsymbol{\cdot}\boldsymbol{q}^{\boldsymbol{-}\boldsymbol{4}}\boldsymbol{+}\boldsymbol{B}\boldsymbol{\cdot}\boldsymbol{P}_{\boldsymbol{core}\boldsymbol{-}\boldsymbol{shell}\boldsymbol{-}\boldsymbol{shell}}\boldsymbol{+}\boldsymbol{bkg}$ | $\chi_{\mathrm{red}}^{2}$ = 1.95 | $\chi_{\mathrm{red}}^{2}$ = 1.25 |
| *A* $\boldsymbol{\in}$ [$\boldsymbol{-\infty}$;$\boldsymbol{\infty}$] | (2.12 ± 0.27) $\cdot$ 10^-11^ | (6.92 ± 0.27) $\cdot$ 10^-11^ |
| *B* $\boldsymbol{\in}$ [$\boldsymbol{-\infty}$;$\boldsymbol{\infty}$] | (1.25 ± 0.10) $\cdot$ 10^-5^ | (7.46 ± 1.02) $\cdot$ 10^-6^ |
| *r_core_* (fixed) [Å] | 53.65 | 53.65 |
| *SLD_core_* (fixed) [Å^-2^] | 6.66 $\cdot$ 10^-6^ | 6.66 $\cdot$ 10^-6^ |
| *SLD_in,1_* (fixed) [Å^-2^] | 0.24 $\cdot$ 10^-6^ | 0.24 $\cdot$ 10^-6^ |
| *SLD_out,1_* (fixed) [Å^-2^] | 5.49 $\cdot$ 10^-6^ | 5.49 $\cdot$ 10^-6^ |
| *d_shell,1_* (fixed) [Å] | 7.00 | 7.00 |
| *SLD_in,2_* (fixed) [Å^-2^] | 5.49 $\cdot$ 10^-6^ | 5.49 $\cdot$ 10^-6^ |
| *SLD_out,2_* (fixed) [Å^-2^] | 6.39 $\cdot$ 10^-6^ | 6.39 $\cdot$ 10^-6^ |
| *SLD_solvent_* (fixed) [Å^-2^] | 6.39 $\cdot$ 10^-6^ | 6.39 $\cdot$ 10^-6^ |
| *d_shell,2_* $\boldsymbol{\in}$ [0.00;25.60] [Å] | 10.40 ± 6.44 | 12.61 ± 6.93 |
| *bkg* $\boldsymbol{\in}$ [$\boldsymbol{-\infty}$;$\boldsymbol{\infty}$] | (2.30 ± 0.03) $\cdot$ 10^-4^ | (2.13 ± 0.03) $\cdot$ 10^-4^ |

**Figure S19: MD simulated atomic radial density profiles before and after E_2_ adsorption.**

The dashed line indicates the mean SPION radius. The profiles are illustrated with transparent MD images. PAC_18_ chains are displayed in grey and PAC_3_Imi+ chains in dark blue with dark blue nitrogen atoms. The E_2_ molecules are shown with a bright green steroid body with red oxygen and white hydrogen atoms. The iron oxide surface is shown with orange iron and red oxygen atoms. Before adsorption, the PAC_18_ molecules organize in bundles and are mostly collapsed on the surface in aqueous environment. Upon adsorption of a saturated amount of E_2_ molecules (2085 per SPION), the SPION model relaxes in favor of hydrophobic segregation from the water phase. Next to the adsorption on PAC_18_, this also leads to intercalation between bundles of the hydrophobic molecules, which in turn are either pushed towards the SPION surface or adopt a more radial orientation. As a consequence, the effective particle hydration radius and, thus, the organic shell thickness increases. This supports both the *d_shell_* increase as well as the lower *SLD_in_* upon adsorption obtained from SANS data modelling via *P_core-shell_*.

# Characterization of TiON model system

**Figure S20: Characterization of unfunctionalized TiONs.**

**a-d**, HR-TEM micrograph (**a**), SAED pattern and radial diffractogram with theoretical peak positions (black lines) as well as indices (**b**, **c**) and N_2_ gas adsorption BET isotherm with derived *SSA*. The TiONs have an average size of 17.6 nm calculated from their *SSA* of 87.3 m^2^ g^-1^ assuming sphericity. This agrees with the micrograph, which also shows strong agglomeration. The data indicate that the TiONs are in the anatase phase, based on the agreement between the observed diffraction peaks and the theoretically expected peaks derived from the structure factor, as indexed and marked accordingly.

**Figure S21: ATR-FTIR spectra of SAM-coated TiONs.**

**a**, **b**, Transmittance ATR-FTIR spectra (**a**) and absorbance spectra segments (**b**) of SAM-coated TiONs with references. Overall, the signals are much smaller than on corresponding SPION systems. However, very weak bands between 3000 and 2840 cm^-1^ (CH_2_ and CH_3_), around 1570 cm^-1^ (CN in imidazolium ring) and between 1200-950 cm^‑1^ (PO) confirm the functionalization with PAC_3_Imi+. For PAC_18_@TiONs, stronger bands around 2955, 2920, 2850 and 1460 cm^-1^ (aliphatic CH_2_ and CH_3_) as well as a convoluted band from 1200-950 cm^-1^ (PO) confirm the functionalization. For PAC_3_Imi+/PAC_18_@TiONs, an intermediate spectrum is obtained, however, PAC_18_ strongly dominates. By referring aliphatic, asymmetrical CH_2_ $\upsilon\left( \mathrm{CH}_{2}^{\mathrm{asym}} \right)$ to aliphatic, asymmetrical CH_3_ $\upsilon\left( \mathrm{CH}_{3}^{\mathrm{asym}} \right)$ vibrational absorbance intensities (**b**), the surface share of both molecules in the binary SAM can be estimated.^2^ In PAC_3_Imi+/PAC_18_@TiONs about 17 % of the binary SAM are PAC_3_Imi+ and about 83 % PAC_18_. The TiON systems spectra in **a** are stretched by a factor of eight relative to the molecule references.

**Figure S22: TGA and derived GD_PA_ of SAM-coated TiONs.**

**a**, **b**, TGA including DTG (**a**) and calculated *GD_PA_* (**b**) of SAM-coated TiONs with unfunctionalized TiONs as reference. All systems show greater mass losses between 150 and 700 °C than unfunctionalized TiONs indicating a substantial organic coverage in all cases. The mass losses are converted to *GD_PA_* estimates assuming the full SSA is available and any stabilizer replaced (PAC_3_Imi+@TiONs: 0.9 ± 0.1 nm^-2^, PAC_3_Imi+/PAC_18_@TiONs: 1.2 ± 0.1 nm^-2^, PAC_18_@TiONs: 1.7 ± 0.1 nm^-2^). While following the same trend as on the SPIONs, the SAMs seem less dense or the available SSA is smaller. Data are shown as mean ± standard deviation from technical *n* = 3 in **a** and **b**.

# Reference ss-NMR spectra and deconvolution

**Figure S23: Normalized reference ^1^H MAS ss-NMR spectra.**

The unfunctionalized TiONs show an intense signal at 5.9 ppm that can be associated to ^1^H of free hydroxyl groups. Signals on the SAM-covered nanoparticles at 0-3 ppm originate mainly from aliphatic protons of PAC_18_, while those at 3-5 ppm are assigned to CH_2_ and CH_3_ protons of PAC_3_Imi+ with minor contributions from residual methanol and water. The superimposing resonances between ca. 6 and 10 ppm correspond to imidazolium ring CH protons and surface hydroxyls including hydroxyl protons of phosphonic acid groups. Note that the ^1^H MAS NMR spectra are normalized with respect to the sample masses. Since more than 93 % of the mass is due to the TiON cores, the intensities of the signals are directly comparable. This is also true for the intensities provided in Figure S24-S29.

**Figure S24: ^1^H MAS ss-NMR spectrum deconvolution of E_2_.**

**a**, **b**, Deconvoluted ^1^H MAS ss-NMR spectrum of E_2_ (**a**) and corresponding signals (**b**). E_2_ exhibits a characteristic intense methyl proton signal at approximately 1.0 ppm [4]. Signals in the range of 1-4 ppm arise from CH_2_ and CH protons of the saturated steroid rings [3,4]. A resonance observed at approximately 4.7 ppm can be attributed to a CH group of a saturated ring bearing a hydroxyl substituent [3]. Aromatic CH protons of the unsaturated ring and hydroxyl protons are observed at higher chemical shifts between 6 and 11 ppm [1,2].

**Figure S25: ^1^H MAS ss-NMR spectrum deconvolution of TiONs.**

**a**, **b**, Deconvoluted ^1^H MAS ss-NMR spectrum of TiONs (**a**) and corresponding signals (**b**). The intense signal observed at 5.9 ppm can be attributed to protons of free hydroxyl groups present on the surface [1].

**Figure S26: ^1^H MAS ss-NMR spectrum deconvolution of PAC_3_Imi+@TiONs.**

**a**, **b**, Deconvoluted ^1^H MAS ss-NMR spectrum of PAC_3_Imi+@TiONs (**a**) and corresponding signals (**b**). Signals in the region between 0 and 3 ppm originate from two coupled CH_2_ groups, one of which is connected to the phosphorus atom [3]. The signal at 4.4 ppm is attributed to CH_2_ and CH_3_ groups attached to the imidazolium ring [2]. Resonances observed between 6 and 10 ppm can be assigned to aromatic CH protons of the imidazolium ring as well as to unbound hydroxyl groups on the TiON surface with possible contributions from residual methanol from the functionalization process and atmospheric water [1]. Superimposed, there are also hydroxyl protons of phosphonic acid groups.

**Figure S27: ^1^H MAS ss-NMR spectrum deconvolution of PAC_18_@TiONs.**

**a**, **b**, Deconvoluted ^1^H MAS ss-NMR spectrum of PAC_18_@TiONs (**a**) and corresponding signals (**b**). In the region between 0 and 3 ppm intense signals are observed and can be assigned to aliphatic CH_2_ and CH_3_ groups originating from the PAC_18_ chain [3]. The signals between ca. 6 and 10 ppm may arise either from unfunctionalized hydroxyl surface sites or from hydroxyl protons of the phosphonic acid groups with possible contributions from residual methanol from the functionalization process and atmospheric water [1,2].

**Figure S28: ^1^H MAS ss-NMR spectrum deconvolution of PAC_3_Imi+/PAC_18_@TiONs.**

**a**, **b**, Deconvoluted ^1^H MAS ss-NMR spectrum of PAC_3_Imi+/PAC_18_@TiONs (**a**) and corresponding signals (**b**). Signals between 0 and 3 ppm can be assigned to aliphatic protons, mainly originating from the PAC_18_ chains [4]. Resonances in the range of 3-5 ppm are primarily attributed to CH_2_ and CH_3_ groups attached to the imidazolium ring of PAC_3_Imi+, with possible contributions from residual methanol from the functionalization process and atmospheric water [3]. Signals between ca. 6 and 10 ppm correspond to aromatic CH protons of the imidazolium ring as well as to unbound hydroxyl groups on the TiON surface and to hydroxyl protons of phosphonic acid groups [1,2].

**Figure S29: ^1^H MAS ss-NMR spectrum deconvolution of PAC_3_Imi+/PAC_18_@TiONs with adsorbed E_2_.**

**a**, **b**, Deconvoluted ^1^H MAS ss-NMR spectrum of PAC_3_Imi+/PAC_18_@TiONs with adsorbed E_2_ (**a**) and corresponding signals (**b**). The spectrum shows the same set of signals as observed for the sample without E_2_. However, a significant increase in signal intensity is observed in the region between 0 and 3 ppm, which corresponds to the most intense proton signals of E_2_ [4]. This increase clearly indicates the adsorption of E_2_.

**Figure S30: Reference ^1^H-^1^H DQ BABA MAS ss-NMR spectra.**

**a**-**f**, ^1^H-^1^H DQ BABA MAS ss-NMR spectra of E_2_ (**a**), TiONs (**b**), PAC_3_Imi+@TiONs (**c**), PAC_18_@TiONs (**d**), PAC_3_Imi+/PAC_18_@TiONs (**e**), and PAC_3_Imi+/PAC_18_@TiONs with adsorbed E_2_ (**f**). In ^1^H-^1^H DQ BABA spectra, DQ correlations appear at F1 frequencies corresponding to the sum of the single-quantum frequencies of the coupled protons. Consequently, homonuclear (AA) correlations give rise to a single autocorrelation peak at (2ωA, ωA), whereas heteronuclear-like (AB) correlations produce two cross-peaks at (ωA + ωB, ωA) and (ωA + ωB, ωB), allowing straightforward identification of coupled proton pairs.^3^ In the spectrum of E_2_ several diagonal signals are observed. In addition, also a proximity correlation can be assigned. The signal at ca. (7.8, 0.8) ppm corresponds to aliphatic and the signal at ca. (7.8, 7.0) ppm c to aromatic protons. This suggests interactions between CH groups of the saturated ring and CH groups of the unsaturated ring. In the spectrum of unfunctionalized TiONs most signals are located close to the diagonal (with some *t_1_* noise). The broad diagonal signal can be assigned to free hydroxyl groups. The spectra of PAC_3_Imi+ and PAC_18_ are dominated by autocorrelation (diagonal) signals and serve as references for signal assignment in PAC_3_Imi+/PAC_18_@TiONs samples. Here, intense aliphatic autocorrelation peaks originate from alkyl chains of PAC_18_ and aliphatic protons of PAC_3_Imi+. Additional diagonal signals are observed for CH_2_ and CH_3_ groups attached to the imidazolium ring between 4 and 5 ppm in SQ dimension. Aromatic CH protons of the imidazolium ring and hydroxyl protons of phosphonic acid groups contribute to signals between ca. 6 and 10 ppm in SQ dimension. **f** is shown for direct comparison, but discussed in Figure 3 in the main manuscript.

# Comprehensive study of functional SAMs on flat AlO_x_ substrates

**Figure S31: Water contact angles and surface energies of SAM-coated AlO_x_ substrates.**

**a**, **b**, Water contact angles (**a**) and surface energies with respective polar and dispersive contributions (**b**) for model SAM-coated AlO_x_ substrates. When PAC_3_Imi+ is deposited, the water contact angle of the surface drops rendering it more hydrophilic as supported by an increase in the polar surface energy contribution. PAC_18_@AlO_x_ shows a strongly increased water contact angle corresponding to its hydrophobic nature giving the surface a much smaller overall surface energy with almost no polar contribution. The binary SAM PAC_3_Imi+/PAC_18_ behaves in between. Data are shown as mean ± standard deviation from technical *n* = 3 in **a**, from which by including triplicates of n-dodecane and ethylene glycol contact angles the surface energies were derived.

**Figure S32: XPS spectra of SAM-coated AlO_x_ substrates.**

**a**-**e**, Narrow-scan XPS spectra of the Al 2s region (**a**), O 1s region (**b**), P 2s region (**c**), C 1s region (**d**) and N 1s region (**e**). Substrate photoelectrons from Al and O are detected from all samples just as P is found on all SAM-coated substrates. C is found strongest on PAC_18_@AlO_x_ and the co-self-assembled PAC_3_Imi+&PAC_18_@AlO_x_ with adventitious C also on the uncoated substrate. N (402 eV) is only detected on PAC_3_Imi+@AlO_x_ and the iteratively self-assembled PAC_3_Imi+/PAC_18_@AlO_x_. The derived atomic shares are given in Table S4.

**Table S4: XPS-estimated atomic shares on SAM-coated AlO_x_ substrates.**

The quantified atomic shares resemble the previous qualitative observations in Figure S32. For quantification of N 1s the signal around 407 eV was not regarded. These shares were used to determine atomic ratios and estimate binary SAM compositions in Table S5.

| Sample | Al 2s  [at%] | O 1s  [at%] | P 2s  [at%] | C 1s  [at%] | N 1s  [at%] |
| --- | --- | --- | --- | --- | --- |
| AlO_x_ substrate | 27.0 | 48.8 | 0.0 | 23.7 | 0.4 |
| PAC_3_Imi+@AlO_x_ | 25.5 | 48.4 | 2.4 | 22.5 | 1.2 |
| PAC_18_@AlO_x_ | 14.7 | 21.1 | 1.8 | 62.2 | 0.2 |
| PAC_3_Imi+/PAC_18_@AlO_x_ | 25.0 | 46.6 | 2.3 | 25.2 | 1.0 |
| PAC_3_Imi+&PAC_18_@AlO_x_ | 16.8 | 26.1 | 1.9 | 54.8 | 0.3 |

**Table S5: XPS-estimated atomic ratios and binary SAM compositions on AlO_x_ substrates.**

By referring atomic shares of P and Al, one can roughly compare SAM grafting densities. PAC_3_Imi+ grafts less dense than PAC_18_, with the iterative binary PAC_3_Imi+/PAC_18_ SAM resembling the PAC_3_Imi+ density and the co-assembled binary PAC_3_Imi+&PAC_18_ SAM resembling the PAC_18_ one. By referring atomic shares of N and P, however, the binary SAM composition can be estimated in relation to the unitary SAMs. PAC_3_Imi+/PAC_18_@AlO_x_ shows about 86 % of PAC_3_Imi+ and 14 % of PAC_18_, while PAC_3_Imi+&PAC_18_@AlO_x_ shows the opposite with 18 % PAC_3_Imi+ and 82 % PAC_18_. Thus, the vast excess of PAC_3_Imi+ in PAC_3_Imi+/PAC_18_@AlO_x_ renders this system a poor model for PAC_3_Imi+/PAC_18_@SPIONs. It has to be mentioned that the expected ideal N/P ratio of two for PAC_3_Imi+@AlO_x_ was not found. However, since ATR-FTIR as well as ss-NMR spectra confirm the presence of the imidazolium moiety and they are generally regarded stable ionic liquids, the reason for the determined N/P ratio remains unclear.

| Sample | P 2s /  Al 2s  [-] | N 1s /  P 2s  [-] | Share  PAC_3_Imi+  [%] | Share  PAC_18_  [%] |
| --- | --- | --- | --- | --- |
| PAC_3_Imi+@AlO_x_ | 0.093 | 0.483 | - | - |
| PAC_18_@AlO_x_ | 0.125 | 0.092 | - | - |
| PAC_3_Imi+/PAC_18_@AlO_x_ | 0.091 | 0.430 | 86 | 14 |
| PAC_3_Imi+&PAC_18_@AlO_x_ | 0.115 | 0.161 | 18 | 82 |

**Figure S33: Representative AFM topography images of SAM-coated AlO_x_ substrates with and without E_2_ present.**

**a**-**d**, AFM topography images of PAC_3_Imi+@AlO_x_ (**a**), PAC_18_@AlO_x_ (**b**), PAC_3_Imi+/PAC_18_@AlO_x_ (**c**) and PAC_3_Imi+&PAC_18_@AlO_x_ (**d**) are comparatively shown in the aqueous solutions without E_2_ (left column) and containing E_2_ (right column). In the left column, **a** was obtained in ultrapure water, and **b**-**d** in ultrapure water containing 1 mM KCl. In the right column, the imaging aqueous solutions contained 1 µM E2 and 1.7 vol% methanol, thus resembling the adsorption conditions. In the left column molecular resolution was achieved revealing row-like features in **a**, **b** and **d** (best visible in **d**). The lattice distances (about 0.5 nm) in **b** and **d** roughly agree with GIWAXS (see Table S6). Thus, those features may represent molecular rows. However, upon immersion in E_2_ and methanol containing solution, molecular resolution is lost, preventing any assessment of E_2_-SAM interaction.

**Figure S34: XRR and Patterson analyses of SAM-coated AlO_x_ substrates before and after E_2_ treatment.**

**a**, **b**, XRR measurements of SAM-coated AlO_x_ substrates before and after treatment with E_2_ (**a**) as well as derived Fourier-transformed and Fresnel-normalized Patterson analysis (**b**). *q_z_* is the vertical value of the scattering vector. Generally, peaks in **b** can be associated with the AlO_x_ coating ($\boldsymbol{\sim}$ 110 Å), PAC_18_ ($\boldsymbol{\sim}$ 20 Å), as well as superimposed higher-order features. The treatment with E_2_ introduces a new feature around 34 Å on the AlO_x_ substrate, PAC_3_Imi+@AlO_x_ and PAC_3_Imi+/PAC_18_@AlO_x_, however, not on the majorly of PAC_18_ containing SAMs, which is counterintuitive to the remediation results. Overall, no new conclusions on the E_2_-SAM interaction can be drawn.

**Figure S35: GIWAXS of SAM-coated AlO_x_ substrates before and after E_2_ treatment.**

**a**, **b**, GIWAXS data of SAM-coated AlO_x_ substrates before and after E_2_ treatment including identified lateral scattering vector values *q_r_* (**a**) and illustration of the hexagonal PAC_18_ lattice represented by blue dots (**b**). Bragg rods can be identified around 1.5 Å^-1^ for PAC_18_@AlO_x_ as well as the majorly of PAC_18_ consisting binary PAC_3_Imi+&PAC_18_@AlO_x_. This corresponds to the 2D hexagonal lattice that PAC_18_ forms as illustrated via a blue anchor group lattice in **b**.^4^ The lattice distance *d_lattice_*, the lattice constant *a_lattice_*, and *GD_PA_* can be calculated from *q_r_* by geometrical considerations as given in Table S6. Thus, PAC_3_Imi+/PAC_18_@AlO_x_ appears non-crystalline, while the co-self-assembled binary PAC_3_Imi+&PAC_18_ SAM shows a crystalline PAC_18_ matrix with PAC_3_Imi+ inclusions_._ The E_2_ treatment yields no structural changes.

**Table S6: Conversion of GIWAXS-determined *q_r_* to lattice constants *d_lattice_* and *a_lattice_* as well as *GD_PA_*.**

The determined lattice parameters match with the row-like features in the AFM topography images. The origin of the increased *GD_PA_* in comparison to values on SPIONs is discussed in Figure S36.

| Sample | *q_r_*  [Å^-1^] | *d_lattice_*  [Å] | *a_lattice_*  [Å] | *GD_PA_*  [nm^-2^] |
| --- | --- | --- | --- | --- |
| PAC_18_@AlO_x_ | 1.49 | 4.22 | 4.87 | 4.87 |
| PAC_3_Imi+&PAC_18_@AlO_x_ | 1.47 | 4.27 | 4.94 | 4.74 |

*Methods*

Boron-doped silicon wafers from Si-Mat – Silicon Materials were coated with AlO_x_ (nominal 10 nm) via atomic layer deposition carried out by the Max Planck Institute for the Science of Light in Erlangen. For functionalization, they were cut to suitable sizes, rinsed with methanol and blow-dried with N_2_. After oxygen plasma activation at 0.2 mbar for 5 min in a plasma chamber Pico from Diener electronic, they were immersed in methanol solutions of the respective phosphonic acid derivative at 0.1 mM for 24 h. For the co-self-assembly approach (PAC_3_Imi+&PAC_18_@AlO_x_), both derivatives were mixed in equal volumetric ratio. For the iterative self-assembly (PAC_3_Imi+/PAC_18_@AlO_x_), the substrates were first immersed in a solution of PAC_3_Imi+ at 0.1 mM for 24 h, rinsed with methanol and immersed again in a PAC_18_ solution at 0.1 mM for 24 h. Afterwards, all samples were rinsed with methanol, blow-dried and cured at 60 °C for 3 min on a heating plate.

Contact angle measurements were carried with about 1 µL of DI water, ethylene glycol, and n-dodecane via the sessile drop method. The angles were measured on both sides and averaged. These measurements were carried out with an OCA 25 running software SCA 20 version 5.0.37 both from DataPhysics Instruments. Per sample and solvent three drops were measured. When the solvent wetted the surface completely and no angle could be measured, a contact angle of 0° was assumed. The surface energies were calculated via the Owens, Wendt, Rabel and Kaelble (OWRK) method.

XPS spectra were measured with an ESCA+ device from Omicron NanoTechnology emitting monochromatic Al Kα X-rays (1486.7 eV) using a hemispherical analyzer at an angle of 30° to the surface plane in an atmosphere smaller than 3$\cdot$10^-10^ mbar without charge neutralization. The binding energies were charge-corrected using the C 1s signal of aliphatic hydrocarbons at 285.0 eV. The spectra were recorded with a step size of 0.1 eV, a pass energy of 20 eV, and a dwell time of 0.5 s. The data was analyzed with CasaXPS version 2.3.16 from Casa Software. Atom percentages were calculated by fitting peaks with Gaussian-Lorentzian sums and Shirley or linear backgrounds.

Amplitude modulation AFM images were recorded with a commercial Asylum Research ES-Cypher AFM system from Oxford Instruments equipped with temperature control and photothermal cantilever actuation. Measurements were performed with Arrow UHF silicon cantilevers from Nanoworld. Each cantilever was calibrated before the measurement using its thermal spectrum^5^ yielding typical flexural stiffnesses of 1.5 ± 0.2 N m^-1^ in solution. All the cantilevers were thoroughly cleaned following a three-steps procedure in order to reduce any potential contamination: The cantilevers were immersed in a bath of ultrapure water for 12 h, followed by isopropanol for 6 h and finally ultrapure water for 60 min.^6,7^ All experiments were performed with the cantilever fully immersed in liquid at 25.0 ± 0.1 °C. Ultrapure water or water with 1 mM KCl were used for all the samples without E_2_. For measurement in the presence of E_2_, we aimed to mimic the magnetic water cleaning conditions: E_2_ was first dissolved in methanol followed by dilution with ultrapure water to obtain a 1 µM aqueous solution with 1 vol% of methanol. Additional methanol caused by pre-dispersion of SPIONs was compensated by increasing the methanol fraction to 1.7 vol%. For a given substrate, measurements were first conducted in ultrapure water followed by the aqueous E_2_ solution. When exchanging solutions, both the sample and the cantilever were thoroughly washed with the estrogen solution (40 times with 100 μL). Imaging was performed in amplitude modulation, photo-thermally driving the cantilever close to its resonant frequency. The phase lag between the driving and tip oscillation could vary freely. Within this mode, the cantilever oscillation amplitude is kept constant and the feedback corrections are used to reconstruct the sample topography.^8^ On every sample at least three areas were probed. First-order flattening, low-pass Butterworth IFFT filtering, and analyses were done using Igor Pro from Wavemetrics and Python. All solvents were purchased from Merck Millipore.

The XRR measurements were performed using a Bruker D8 diffractometer equipped with a Cu radiation source (about 1.54 Å), a Ni filter, a Goebel mirror, a 0.2 mm slit, and an extra-long scatter guard. The Ni filter was used to reduce Cu Kβ radiation. The Goebel mirror monochromatized to Kα and parallelized the X-ray beam. The slit was used to shape the beam to a specific size and the scatter guard to reduce the background. As detector an Eiger2 R 500K from Dectris was used in 0D mode with a pixel height of 3 pixels (fully capturing the 0.2 mm beam) and a width that corresponds to the width of the sample (1 pixel = 75 µm x 75 µm). Before the XRR measurements, beam, height and surface alignment steps were performed until no further changes were detected. For every sample first an overview scan was performed followed by an automated measurement from 0° to 5° incident angle. The angular ranges, counting times and step widths were automatically set by the Diffrac.Wizard software from Bruker (see **Table S7**). Backgrounds at an offset in the angle of incidence by 0.15° were subtracted from the acquired intensities before calculating the XRR, which were normalized to the maximum recorded intensity. Additionally, a simple footprint correction assuming a box shaped beam was applied to all curves. For the Patterson analyses, the XRRs were divided by the respective Fresnel reflectivity of the substrate and Fourier-transformed between 0.093 and 0.5 Å^-1^. A critical value of the scattering vector of 0.0317 Å^‑1^ (Cu Kα X-rays) was assumed for the substrate.^9^ To adsorb E_2_ on samples, they were immersed for 10 min in an about 1 µM aqueous E_2_ solution (prepared similarly as for the AFM measurements), followed by rinsing with DI water to remove unbound species and blow-drying.

**Table S7: XRR measurement angular ranges, counting times and step widths.**

Exact values for all XRR samples as automatically set by Diffrac.Wizard software from Bruker. Inc stands for step width (increment).

| AlO_x_ substrate | AlO_x_ substrate  + E_2_ | PAC_3_Imi+@AlO_x_ | PAC_3_Imi+@AlO_x_  + E_2_ | PAC_18_@AlO_x_ | PAC_18_@AlO_x_  + E_2_ | PAC_3_Imi+/PAC_18_@AlO_x_ | PAC_3_Imi+/PAC_18_@AlO_x_  + E_2_ | PAC_3_Imi+&PAC_18_@AlO_x_ | PAC_3_Imi+&PAC_18_@AlO_x_  + E_2_ |
| --- | --- | --- | --- | --- | --- | --- | --- | --- | --- |
| 0°-2.5°; 1 s/step; 0.005° Inc | 0°-2.5°; 1 s/step; 0.01° Inc | 0°-2.5°; 1 s/step; 0.01° Inc | 0°-2.5°;  1 s/step; 0.01° Inc | 0°-2.5°;  1 s/step; 0.01° Inc | 0°-2.5°;  1 s/step; 0.01° Inc | 0°-2.5°;  1 s/step; 0.01° Inc | 0°-2.5°;  1 s/step; 0.01° Inc | 0°-2.5°;  1 s/step; 0.01° Inc | 0°-2.5°;  1 s/step; 0.01° Inc |
| 2.5°-3.95°; 4 s/step; 0.005°Inc | 2.5°-3.95°: 4 s/step; 0.01° Inc | 2.5°-3.95°; 4 s/step; 0.01° Inc | 2.5°-3.95°; 4 s/step; 0.01° Inc | 2.5°-3.95°; 4 s/step; 0.01° Inc | 2.5°-3.95°; 4 s/step; 0.01° Inc | 2.5°-3.95°; 4 s/step; 0.01° Inc | 2.5°-3.95°; 4 s/step; 0.01° Inc | 2.5°-3.95°; 4 s/step; 0.01° Inc | 2.5°-3.95°; 4 s/step; 0.01° Inc |
| 3.95°-5°; 8 s/step; 0.005° Inc | 3.95°-4.99°; 10 s/step; 0.02° Inc | 3,95°-5°; 8 s/step; 0.05° Inc | 3.95°-4.99°; 10 s/step; 0.02° Inc | 3.95°-4.99°; 10 s/step; 0.02° Inc | 3.95°-4.99°; 10 s/step; 0.02° Inc | 3.95°-4.99°; 10 s/step; 0.02° Inc | 3.95°-4.99°; 10 s/step; 0.02° Inc | 3.95°-4.99°; 10 s/step; 0.02° Inc | 3.95°-4.99°; 10 s/step; 0.02° Inc |
| 5°-6°; 12 s/step; 0,005° Inc | 4.99°-5.99°; 80 s/step; 0.04° Inc | 5°-6°; 12 s/step; 0.01° Inc | 4.99°-5.99°; 80 s/step; 0.04° Inc | 4.99°-5.99°; 80 s/step; 0.04° Inc | 4.99°-5.99°; 80 s/step; 0.04° Inc | 4.99°-5.99°; 80 s/step; 0.04° Inc | 4.99°-5.99°; 80 s/step; 0.04° Inc | 4.99°-5.99°; 80 s/step; 0.04° Inc | 4.99°-5.99°; 80 s/step; 0.04° Inc |
| 6°-7.365°; 40 s/step; 0,005° Inc | 5.99°-6.99°; 160 s/step; 0.05° Inc | 6° - 7°;  40 s/step; 0.01° Inc | 5.99°-6.99°; 160 s/step; 0.05° Inc | 5.99°-7.99°; 40 s/step; 0.05° Inc | 5.99°-6.99°; 160 s/step; 0.05° Inc | 5.99°-6.99°; 160 s/step; 0.05° Inc | 5.99°-6.99°; 160 s/step; 0.05° Inc | 5.99°-7.99°; 160 s/step; 0.05° Inc | 5.99°-6.99°; 160 s/step; 0.05° Inc |
|  | 7°-8°;  200 s/step;  0.05° Inc | 7°-8.5°; 80 s/step; 0.01° Inc | 7°-8°; 200 s/step; 0.05° Inc |  | 7°-8°; 200 s/step; 0.05° Inc | 7°-8°; 200 s/step; 0.05° Inc | 7°-8°; 200 s/step; 0.05° Inc | 8°-9.99°; 200 s/step; 0.07° Inc | 7°-8°; 200 s/step; 0.05° Inc |
|  | 8°-10.03°; 300 s/step; 0.07° Inc | 8.5°-10°; 120 s/step; 0.01° Inc | 8°-10.03°; 300 s/step; 0.07° Inc |  | 8°-10.03°; 300 s/step; 0.07° Inc | 8°-10.03°; 300 s/step; 0.07° Inc | 8°-10.03°; 300 s/step; 0.07° Inc |  | 8°-10.03°; 300 s/step; 0.07° Inc |

GIWAXS measurements were performed with the same device and detector. As the incident beam optic, we used a polycapillary optic, which produces a high flux quasi-circular and “parallel” beam of about 4 mm diameter with a nominal divergence of about 0.25°. Additionally, we utilized a circular 2 mm universal beam collimator to define the beam size. The incident angle was set to 0.2°, *i.e.*, below the critical angle of the substrate. The relevant critical angles at this energy are 0.23° and 0.29° for silicon and alumina, respectively. In order to minimize air scattering, the samples were housed in a custom-made vacuum chamber with Kapton windows, which was maintained under continuous vacuum using a Pfeiffer membrane vacuum pump 015-2. Additionally, a beam stop inside the chamber was used to block the specularly reflected and transmitted beam. Between the sample and the detector 0.5° axial Soller slits were positioned. The detector was configured with a 300 x 300 pixel region of interest (roughly corresponding to the Soller slit opening) and placed on the in-plane detector arm at a sample-to-detector distance of 312.5 mm. The angular resolution of this setup is about 0.33° (*i.e.*, full width half maximum) as determined with a primary beam scan. Measurements were performed for in-plane (non-coplanar) scattering angles between 10° and 32° with a step size of 0.176° and an exposure time of 700 s per step. The collected images were subsequently processed in Python using SciPy^10^ and numpy. A 125 x 150 pixel region of interest excluding edge effects from the Soller slits was chosen and integrated along the in-plane direction for each image, mimicking a 1D detector with intensity distribution along *q_z_*. Subsequently, these 1D arrays were stitched together by assigning a *q_r_* value to each scattering angle 2*θ* (for each array) to produce a 2D scattering pattern in *q_z_/q_r_*-space. Integration along *q_z_* was then performed to obtain *I(q_r_)* (excluding the region below the horizon, *i.e.*, *q_z_*⪅0). The data were analyzed by fitting a Gaussian with linear background to reveal the peak position, peak width, and peak area. The lattice distance *d_lattice_*, the lattice constant *a_lattice_*, and *GD_PA_* can be calculated from *q_r_* by geometrical considerations according to **Equations** **S1-S3**. To adsorb E_2_, the samples were treated similar as for XRR.

$$d_{lattice}=\frac{2\pi}{q_{r}}$$

(S1)

$$a_{lattice}=\frac{d_{lattice}}{\cos\left( 30^{\circ} \right)}$$

(S2)

$${GD}_{PA}=\frac{2}{\sqrt{3}\cdot a_{lattice}^{2}}$$

(S3)

# MD simulation support, coordination analyses, and methodology

**Figure S36: A simulated SPION with varying *GD_PA_* values of PAC_18_.**

**a**, **b**, **c**, SPION (**a**) covered with PAC_18_ at *GD_PA_* of 2.21 nm^-2^ (**b**) and 4.57 nm^-2^ (**c**) in vacuum. The illustration in **b** is similar to the TGA-derived *GD_PA_* on SPIONs and shows non-saturated grafting, while **c** is similar to the GIWAXS-derived *GD_PA_* on flat AlO_x_ substrates and appears saturated with hexagonal crystallinity. In reality, the SPIONs are covered in saturation, too (experimentally determined by phosphonic acid concentration series). The TGA-derived values base on the assumption that the whole BET-derived *SSA* is available, which results in coverage below saturation as shown in **b**. The uncovered surface is likely blocked due to SPION-SPION agglomeration. We decided to run the simulations close to TGA-derived values as the phosphonic acid available *SSA* was experimentally inaccessible. PAC_18_ chains are displayed in grey with yellow phosphorus, red oxygen, and white hydrogen atoms. The iron oxide surface is shown with orange iron and red oxygen atoms.

*General setup*

All simulations were performed using LAMMPS^11^ accelerated with the GPU package.^12–17^ Each run was performed with 3D periodic boundary conditions and a damp-shifted-forces potential with a damping factor of *α* = 0.2 and a cutoff radius of *r* = 12 Å.^18^ A Nosé-Hoover thermostat (*τ* = 0.5 ps) and, in NpT simulations, a Nosè-Hoover barostat (*τ* = 1.0 ps) were used to achieve temperature and isotropic pressure control.^19^ Velocity Verlet time integration was realized using a timestep of 1.0 fs.^20^ The initial structures of the organic molecules, namely PAC_3_Imi+, PAC_18_, and the estrogen derivatives E_1_, EE_2_, E_2_ and E_3_ were created using Schrödinger’s Maestro 2022-1.^21^ Next, Gaussian16^22^ was used to perform DFT geometry optimizations using B3LYPT/631-G(d). The electrostatic potentials of the molecules in the gas phase were used to obtain partial charges via RESP fit using antechamber.^23–25^ TIP3P-CHARMM^26^ parameters were used to describe flexible water molecules. The force field parameters for maghemite were taken from Erlebach *et al.*^27^ All remaining non-bonded interactions were modelled using 12-6 Lennard-Jones potentials, derived from GAFF^28^ for organics and maghemite oxygen atoms as well as from Li *et al.* for bromide and maghemite iron atoms, respectively.^29,30^ Lorentz-Berthelot combining rules were applied.

*SPION generation*

The maghemite crystal structure was adopted from the ICSD database.^31^ As a starting point, a crystalline cube with edges of length *a* = 10 nm was created via Schrödinger Materials 2022-1.^32^ To obtain a relaxed nanoparticle structure, the model was subjected to melting and re-crystallization whilst keeping the inner core (cube of 1 nm dimensions) fixed. Within 10 ns, the system was heated up to temperature *T* = 2000 K and then quenched to *T* = 300 K within 70 ns. Different cooling rates were probed and convergence of total energy assessed within < 5 % margins. The resulting SPION model reflects a facetted maghemite particle, which resembles a roughly spherical shape with an approximate radius of 6.3 nm.

*SPION functionalization and relaxation*

The golden spiral algorithm was used to place 160 PAC_3_Imi+ (with Br^-^) and 639 PAC_18_ molecules within 1.5-10 Å around the particle, mimicking the experimentally determined SAM composition (20:80 ratio) and an *GD_PA_* of 1.66 nm^-2^. By applying a harmonic potential wall with *r_start_* = 80 Å and *r_end_* = 63.2 Å, which only acted on the Phosphorus atoms, the newly placed molecules were constrained to the surface. Using NVT dynamics in vacuum, a cubic box with 24 nm edge length was first heated up to *T* = 450 K within 1 ns, where *T* was held constant for 10 ns, followed by quenching to *T* = 300 K within 3 ns. Finally, the harmonic wall potential was switched off and the system was allowed to equilibrate for additional 3 ns at room temperature without artificial bias.

The functionalized nanoparticle was then immersed in 414,949 molecules of TIP3P water. We first relaxed the water phase whilst keeping the SPION fixed. For this, the system was heated up from *T* = 100 K to 300 K within 1 ns using NpT dynamics at atmospheric pressure and then propagated for further 2 ns at ambient conditions. Afterwards, all atoms except the nanoparticle core (*r* < 5.8 nm) were subjected to NpT dynamics at ambient conditions for 15 ns.

*Estrogen loading*

To account for the abundance of possible estrogen-SAM interactions, we mimicked SPION immersion in an artificial melt of E_2_ molecules. After relaxation in analogy to the procedure described above, we depicted the contact shell using radial distance delimiters of *r_inner_* = 80 Å and *r_outer_* = 90 Å, respectively. On this basis, a total of 2085 E_2_ molecules was taken as a proxy for possible binding motifs. The solvation of the heavily loaded SPION models was implemented by means of 383,435 water molecules relaxed in analogy to the unloaded SPION described above. Density profiles of the SPION-water and SPION-E_2_-water systems are shown in Figure S19. From the heavily loaded SPION model we then moved to a more realistic, moderately loaded SPION model. For this, 10 E_2_ molecules were randomly selected whilst dismissing all other absorbates. For the sake of comparability, the same starting poses were used for all E_1_, EE_2_, and E_3_, too. For each model, three independent simulation systems were created in this manner.

*Analyses of estrogen-loaded SPION systems*

Each SPION loaded with 10 estrogen molecules was subjected to analogous relaxation procedures as described above. Each system underwent NpT dynamics at ambient conditions for 35 ns, omitting the first 5 ns from analyses to ensure equilibration. For EE_2_, two additional triplicates were performed at *T* = 500 K (5 ns) and *T* = 750 K (35 ns), respectively, to sample equilibria between SPION-bound and freely solvated estrogen molecules. These states were distinguished by a SPION-estrogen minimum atom distance delimiter of 2.5 Å. At *T* = 500 K, no unbinding was observed, but at *T* = 750 K the equilibrium was shifted towards the freely diffusing estrogen molecules.

Minimum distances of any estrogen atom to maghemite or PAC_18_, as well as the angle between the SPION center of mass, (COM_SPION_) the oxygen-bound aromatic carbon atom (C_aromatic_) and the oxygen-bound aliphatic carbon (C_aliphatic_) of the steroid backbone (COM_SPION_– C_aromatic_ – C_aliphatic_) were monitored every 0.1 ns. Additionally, the radial distribution function *g(r)*, the per-molecule distance-dependent number of coordinated atoms *N_coord_(r)*, as well as hydrogen bond and C contact numbers were computed for potentially interacting atom groups (see **Figure S37**-**Figure S44** and **Table S8**) using the RDF class of MDAnalysis.^33,34^ On this basis, we chose the first local minimum for interactions featuring estrogen hydroxyl or carbonyl groups at *r =* 2.5 Å as the distance delimiter. For hydrophobic interactions with water, we identified a C-O distance of *r =* 4.0 Å as cutoff, discriminating well between SPION-bound and freely solvated estrogen molecules, as well as between non-intercalated (type I/II) and intercalated (type III) binding motifs, respectively. From screening of the data, we argue that type III leads to a slight loss in hydrogen bonding with water (see Figure S37-Figure S38), which can occasionally be replaced with phosphonic acid or iron oxide interactions on the SPION surface (see Figure S39-Figure S42). Additionally, a significant loss of unfavorable water contacts can be identified for molecules in type III association, which is replaced with contacts to PAC_18_ (see Figure S43-Figure S44). For E_1_, E_2_, EE_2_ and E_3,_ all poses with less than 5 water molecules in hydrophobic contact and less than 4, 6, 4 and 6 hydrogen bonds to water, respectively, as well as those with less than 10 hydrophobic contacts, but with at least one hydrogen bond to O^2-^ were identified as type III. A molecule was solely recognized as type III if it matched these criteria in at least 20 % of the analyzed time frames.

*Thermodynamic analyses*

For each of the ten adsorbed estrogens, percentages for both SPION-bound and freely dispersed states were calculated from the simulation trajectories leading to average binding probabilities *p_bound_* and, in turn, probabilities for free estrogen *p_free_* = 1-*p_bound_.* These values were used to calculate the differences in free energy between the bound and free state as an association free energy Δ*G* shown in **Equation S4**, where *k_B_* is the Boltzmann constant:

$$\Delta G=-k_{B}T\cdot\ln\left( \frac{p_{bound}}{p_{free}} \right)$$

(S4)

The resulting Gibbs free energy values at *T* = 750 K are positive since the equilibrium is shifted towards the unbound state. However, we used this Δ*G* to calculate the temperature-independent difference in chemical potential Δ*μ* by subtracting a correction term for the entropy of dilution shown in **Equation S5**, where *V_bound_* is the volume accessible to bound estrogens and *V_free_* the free volume:

$$\Delta G=\Delta\mu-k_{B}T\cdot\ln\left( \frac{V_{bound}}{V_{free}} \right)$$

(S5)

While gaining thermodynamic stability from interactions with the SAM, bound molecules are restricted in their vibrational, rotational, and translational degrees of freedom. However, compared to the entropy of dilution, their contribution to Δ*μ* is considered as rather insignificant. The binding volume (*V_bound_* = 219 nm^3^) can be approximated as a spherical shell around the nanoparticle with the inner radius *r_in_* = 8.227 nm, obtained by adding 0.1 nm for C-H bonds to the radius obtained from radial density analysis of the terminal PAC_18_ carbons. The outer radius can be calculated as *r_out_ = r_in_ + d,* using the previously introduced binding delimiter *d =* 2.5 Å. For NpT simulations, the mean volume was determined via Gaussian distributions of each box volume (around 12,610 nm^3^), while for NVT simulations the volume *V_box_* = 13,824 nm^3^ is known. Thus, the volume accessible to free estrogen molecules is calculated via *V_free_ = V_box_ – V_out_,* using the spherical volume of radius *r_out_*. Thus, while the free volume for NpT simulations at *T* = 750 K is simulation-specific, the bound volume remains the same at *V_bound_* = 219 nm^3^ and the free volume for NVT simulations was calculated as *V_free_* = 13,605 nm^3^.

**Figure S37: Radial distribution functions *g(r)* and numbers of coordinated atoms *N_coord_(r)*** **for O_estrogen_-H_water_ interaction.**

**a**,**b**, Radial distribution functions (**a**) and numbers of coordinated atoms (**b**) averaged over all O_estrogen_-H_water_ distances for estrogens in solution (solid), identified as non-intercalated, *i.e.*, type I/II (dashed), and identified as intercalated, *i.e.*, type III (dotted). The dashed black lines at *r* = 2.5 Å mark the cutoff radius for hydrogen bond identification. Statistics according to distance delimiters are compared to manually selected time frames for well-defined scenarios showing type III molecules and the remaining time frames for all estrogen molecules in type I/II adsorption.

**Figure S38: Radial distribution functions *g(r)* and numbers of coordinated atoms *N_coord_(r)*** **for OH_estrogen_-O_water_ interaction.**

**a**,**b**, Radial distribution functions (**a**) and numbers of coordinated atoms (**b**) averaged over all OH_estrogen_-O_water_ distances for estrogens in solution (solid), identified as non-intercalated, *i.e.*, type I/II (dashed), and identified as intercalated, *i.e.*, type III (dotted). The dashed black lines at *r* = 2.5 Å mark the cutoff radius for hydrogen bond identification. Statistics according to distance delimiters are compared to manually selected time frames for well-defined scenarios showing type III molecules and the remaining time frames for all estrogen molecules in type I/II adsorption.

**Figure S39: Radial distribution functions *g(r)* and numbers of coordinated atoms *N_coord_(r)*** **for O_estrogen_-H_PA_ interaction.**

**a**,**b**, Radial distribution functions (**a**) and numbers of coordinated atoms (**b**) averaged over all O_estrogen_-H_PA_ distances for estrogens identified as non-intercalated, *i.e.*, type I/II (solid), and identified as intercalated, *i.e.*, type III (dashed). The dashed black lines at *r* = 2.5 Å mark the cutoff radius for hydrogen bond identification. Statistics according to distance delimiters are compared to manually selected time frames for well-defined scenarios showing type III molecules and the remaining time frames for all estrogen molecules in type I/II adsorption.

**Figure S40: Radial distribution functions *g(r)* and numbers of coordinated atoms *N_coord_(r)*** **for OH_estrogen_-O_PA_ interaction.**

**a**,**b**, Radial distribution functions (**a**) and numbers of coordinated atoms (**b**) averaged over all OH_estrogen_-O_PA_ distances for estrogens identified as non-intercalated, *i.e.*, type I/II (solid), and identified as intercalated, *i.e.*, type III (dashed). The dashed black lines at *r* = 2.5 Å mark the cutoff radius for hydrogen bond identification. Statistics according to distance delimiters are compared to manually selected time frames for well-defined scenarios showing type III molecules and the remaining time frames for all estrogen molecules in type I/II adsorption.

**Figure S41: Radial distribution functions *g(r)* and numbers of coordinated atoms *N_coord_(r)*** **for O_estrogen_-Fe^3+^ interaction.**

**a**,**b**, Radial distribution functions (**a**) and numbers of coordinated atoms (**b**) averaged over all O_estrogen_-Fe^3+^ distances for estrogens identified as non-intercalated, *i.e.*, type I/II (solid), and identified as intercalated, *i.e.*, type III (dashed). The dashed black lines at *r* = 2.5 Å mark the cutoff radius for bond identification. Statistics according to distance delimiters are compared to manually selected time frames for well-defined scenarios showing type III molecules and the remaining time frames for all estrogen molecules in type I/II adsorption.

**Figure S42: Radial distribution functions *g(r)* and numbers of coordinated atoms *N_coord_(r)*** **for OH_estrogen_-O^2-^ interaction.**

**a**,**b**, Radial distribution functions (**a**) and numbers of coordinated atoms (**b**) averaged over all OH_estrogen_-O^2-^ distances for estrogens identified as non-intercalated, *i.e.*, type I/II (solid), and identified as intercalated, *i.e.*, type III (dashed). The dashed black lines at *r* = 2.5 Å mark the cutoff radius for hydrogen bond identification. Statistics according to distance delimiters are compared to manually selected time frames for well-defined scenarios showing type III molecules and the remaining time frames for all estrogen molecules in type I/II adsorption.

**Figure S43: Radial distribution functions *g(r)* and numbers of coordinated atoms *N_coord_(r)*** **for C_estrogen_-O_water_ interaction.**

**a**,**b**, Radial distribution functions (**a**) and numbers of coordinated atoms (**b**) averaged over all C_estrogen_-O_water_ distances for estrogens in solution (solid), identified as non-intercalated, *i.e.*, type I/II (dashed), and identified as intercalated, *i.e.*, type III (dotted). The dashed black lines at *r* = 4 Å mark the cutoff radius for hydrophobic contact identification. Statistics according to distance delimiters are compared to manually selected time frames for well-defined scenarios showing type III molecules and the remaining time frames for all estrogen molecules in type I/II adsorption.

**Figure S44: Radial distribution functions *g(r)* and numbers of coordinated atoms *N_coord_(r)*** **for C_estrogen_-C_PAC18_ interaction.**

**a**,**b**, Radial distribution functions (**a**) and numbers of coordinated atoms (**b**) averaged over all C_estrogen_-C_PAC18_ distances for estrogens identified as non-intercalated, *i.e.*, type I/II (solid), and identified as intercalated, *i.e.*, type III (dashed). The dashed black lines at *r* = 4 Å mark the cutoff radius for hydrophobic contact identification. Statistics according to distance delimiters are compared to manually selected time frames for well-defined scenarios showing type III molecules and the remaining time frames for all estrogen molecules in type I/II adsorption.

**Table S8: Coordination analyses of estrogen derivatives on a PAC_3_Imi+ and PAC_18_ binary SAM.**

Coordination number intervals are provided for estrogen molecules bound to the SPION model in aqueous solution (of type I/II as well as of type III) contrasted to solute coordination in bulk water.

| Estrogen derivative | E_1_ | EE_2_ | E_2_ | E_3_ |
| --- | --- | --- | --- | --- |
| H-bonds O_estrogen_ - H_water_  (SPION / bulk water) | **I/II:** 2-3 / 3-4  **III:** 1-2 / 3-4 | **I/II:** 2-3 / 2-3  **III:** 1-2 / 2-3 | **I/II:** 2-3 / 2-3  **III:** 1-2 / 2-3 | **I/II:** 3 / 3-4  **III:** 2-3 / 3-4 |
| H-bonds OH_estrogen_ - O_water_  (SPION / bulk water) | **I/II:** 0-1 / 0-1  **III:** 0-1 / 0-1 | **I/II:** 1-2 / 1-2  **III:** 1-2 / 1-2 | **I/II:** 1-2 / 1-2  **III:** 1-2 / 1-2 | **I/II:** 2-3 / 2-3  **III:** 2-3 / 2-3 |
| H-bonds O_estrogen_ - H_PA_  (SPION / bulk water) | **I/II:** 0 / 0  **III:** 0 / 0 | **I/II:** 0 / 0  **III:** 0 / 0 | **I/II:** 0 / 0  **III:** 0 / 0 | **I/II:** 0-1 / 0-1  **III:** 0-1 / 0-1 |
| H-bonds OH_estrogen_ - O_PA_  (SPION / bulk water) | **I/II:** 0-1 / 0  **III:** 0-1 / 0 | **I/II:** 0-1 / 0  **III:** 0-1 / 0 | **I/II:** 0-1 / 0  **III:** 0-1 / 0 | **I/II:** 0-1 / 0  **III:** 0-1 / 0 |
| H-bonds OH_estrogen_ - O^2-^  (SPION / bulk water) | **I/II:** 0 / 0  **III:** 0-1 / 0 | **I/II:** 0 / 0  **III:** 0-1 / 0 | **I/II:** 0 / 0  **III:** 0-1 / 0 | **I/II:** 0 / 0  **III:** 0-1 / 0 |
| Contacts C_estrogen_ - O_water_  (SPION / bulk water) | **I/II:** 10-11 / 23-24  **III:** 5-6 / 23-24 | **I/II:** 12-13 / 26-27  **III:** 5-6 / 26-27 | **I/II:** 11-12 / 23-24  **III:** 4-5 / 23-24 | **I/II:** 10-11 / 23-24  **III:** 4-5 / 23-24 |

# References

1.	Müller, L. *et al.* Tailorable nanoparticles for magnetic water cleaning of polychlorinated biphenyls. *Small Methods* **10**, 2500537 (2026).

2.	Voß, J., Müller, L., Park, H. & Halik, M. Deciphering mixed self-assembled monolayers on metal oxide nanoparticle surfaces. *ACS Appl. Mater. Interfaces* **17**, 46320–46329 (2025).

3.	Brown, S. P. & Spiess, H. W. Advanced solid-state NMR methods for the elucidation of structure and dynamics of molecular, macromolecular, and supramolecular systems. *Chem. Rev.* **101**, 4125–4156 (2001).

4.	Schmaltz, T. *Molecular order in p- and n-type self-assembled monolayer field-effect transistors*. (Friedrich-Alexander-Universität Erlangen-Nürnberg, 2014).

5.	Butt, H. J. & Jaschke, M. Calculation of thermal noise in atomic force microscopy. *Nanotechnology* **6**, 1–7 (1995).

6.	Cafolla, C. & Voïtchovsky, K. Lubricating properties of single metal ions at interfaces. *Nanoscale* **10**, 11831–11840 (2018).

7.	Cafolla, C., Voïtchovsky, K. & Payam, A. F. Simultaneous quantification of Young’s modulus and dispersion forces with nanoscale spatial resolution. *Nanotechnology* **34**, 505714 (2023).

8.	García, R. & Pérez, R. Dynamic atomic force microscopy methods. *Surf. Sci. Rep.* **47**, 197–301 (2002).

9.	Daillant, J. & Gibaud, A. *X-Ray and Neutron Reflectivity: Principles and Applications*. (Springer Berlin, Heidelberg, 1999).

10.	Virtanen, P. *et al.* SciPy 1.0: fundamental algorithms for scientific computing in Python. *Nat. Methods* **17**, 261–272 (2020).

11.	Thompson, A. P. *et al.* LAMMPS - a flexible simulation tool for particle-based materials modeling at the atomic, meso, and continuum scales. *Comput. Phys. Commun.* **271**, 108171 (2022).

12.	Nguyen, T. D. GPU-accelerated Tersoff potentials for massively parallel Molecular Dynamics simulations. *Comput. Phys. Commun.* **212**, 113–122 (2017).

13.	Nguyen, T. D. & Plimpton, S. J. Accelerating dissipative particle dynamics simulations for soft matter systems. *Comput. Mater. Sci.* **100**, 173–180 (2015).

14.	Nikolskiy, V. & Stegailov, V. GPU Acceleration of four-site water models in LAMMPS. in *Parallel Computing: Technology Trends* (eds. Foster, I., Joubert, G. H., Kucera, L., Nagel, W. E. & Peters, F.) vol. 36 565–573 (IOP Press, 2020).

15.	Brown, W. M., Kohlmeyer, A., Plimpton, S. J. & Tharrington, A. N. Implementing molecular dynamics on hybrid high performance computers – Particle - particle particle - mesh. *Comput. Phys. Commun.* **183**, 449–459 (2012).

16.	Brown, W. M., Wang, P., Plimpton, S. J. & Tharrington, A. N. Implementing molecular dynamics on hybrid high performance computers – short range forces. *Comput. Phys. Commun.* **182**, 898–911 (2011).

17.	Brown, W. M. & Yamada, M. Implementing molecular dynamics on hybrid high performance computers — Three-body potentials. *Comput. Phys. Commun.* **184**, 2785–2793 (2013).

18.	Zahn, D., Schilling, B. & Kast, S. M. Enhancement of the Wolf damped Coulomb potential: Static, dynamic, and dielectric properties of liquid water from molecular simulation. *J. Phys. Chem. B* **106**, 10725–10732 (2002).

19.	Shinoda, W., Shiga, M. & Mikami, M. Rapid estimation of elastic constants by molecular dynamics simulation under constant stress. *Phys. Rev. B* **69**, 134103 (2004).

20.	Tuckerman, M. E., Alejandre, J., López-Rendón, R., Jochim, A. L. & Martyna, G. J. A Liouville-operator derived measure-preserving integrator for molecular dynamics simulations in the isothermal – isobaric ensemble. *J. Phys. A. Math. Gen.* **39**, 5629–5651 (2006).

21.	Schrödinger LLC. *Schrödinger Release 2022-1: Maestro*. (2022).

22.	Frisch, M. J. *et al.* *Gaussian 16, Revision A.03*. (2016).

23.	Case, D. A. *et al.* *Amber 2022*. (2022).

24.	Case, D. A. *et al.* AmberTools. *J. Chem. Inf. Model.* **63**, 6183–6191 (2023).

25.	Case, D. A. *et al.* Recent developments in Amber biomolecular simulations. *J. Chem. Inf. Model.* **65**, 7835–7843 (2025).

26.	MacKerell, A. D. *et al.* All-atom empirical potential for molecular modeling and dynamics studies of proteins. *J. Phys. Chem. B* **102**, 3586–3616 (1998).

27.	Erlebach, A., Kurland, H., Grabow, J. & Müller, F. A. Structure evolution of nanoparticulate Fe2O3. *Nanoscale* **7**, 2960–2969 (2015).

28.	Wang, J., Wolf, R. M., Caldwell, J. W., Kollman, P. A. & Case, D. A. Development and testing of a general Amber force field. *J. Comput. Chem.* **25**, 1157–1174 (2004).

29.	Li, P., Song, L. F. & Merz, K. M. Parameterization of highly charged metal ions using the 12-6-4 LJ-type nonbonded model in explicit water. *J. Phys. Chem. B* **119**, 883–895 (2015).

30.	Li, P., Song, L. F. & Merz, K. M. Systematic parameterization of monovalent ions employing the nonbonded model. *J. Chem. Theory Comput.* **11**, 1645–1657 (2015).

31.	American Mineralogist Crystal Structure Database. https://rruff.geo.arizona.edu/AMS/minerals/Maghemite (2025). (accessed February 4, 2026).

32.	Schrödinger LLC. *Schrödinger Release 2022-1: Materials Suite*. (2022).

33.	Michaud-Agrawal, N., Denning, E. J., Woolf, T. B. & Beckstein, O. MDAnalysis : A toolkit for the analysis of molecular dynamics simulations. *J. Comput. Chem.* **32**, 2319–2327 (2011).

34.	Gowers, R. J. *et al.* MDAnalysis : A Python package for the rapid analysis of molecular dynamics simulations. in *Proc. of the 15th Python in Science Conf.* 98–105 (2016).
